# Mechanisms of metabolic adaptation in the duckweed *Lemna gibba*: an integrated metabolic, transcriptomic and flux analysis

**DOI:** 10.1186/s12870-023-04480-9

**Published:** 2023-10-03

**Authors:** Hai Shi, Evan Ernst, Nicolas Heinzel, Sean McCorkle, Hardy Rolletschek, Ljudmilla Borisjuk, Stefan Ortleb, Robert Martienssen, John Shanklin, Jorg Schwender

**Affiliations:** 1 Biology Department, Brookhaven National Laboratory, Upton, NY 11973 USA; 2 Cold Spring Harbor Laboratory, 1 Bungtown Rd, Cold Spring Harbor, NY 11724 USA; 3 Howard Hughes Medical Institute, Cold Spring Harbor Laboratory, 1 Bungtown Road, Cold Spring Harbor, NY 11724 USA; 4 Department of Molecular Genetics, Leibniz Institute of Plant Genetics and Crop Plant Research, D–06466 Seeland OT Gatersleben, Germany; 5 Brookhaven National Laboratory, Computational Science Initiative, Upton, NY 11973 USA

**Keywords:** Duckweeds, *Lemna gibba*, Metabolic flux analysis, Metabolome analysis, Transcriptome analysis

## Abstract

**Background:**

Duckweeds are small, rapidly growing aquatic flowering plants. Due to their ability for biomass production at high rates they represent promising candidates for biofuel feedstocks. Duckweeds are also excellent model organisms because they can be maintained in well-defined liquid media, usually reproduce asexually, and because genomic resources are becoming increasingly available. To demonstrate the utility of duckweed for integrated metabolic studies, we examined the metabolic adaptation of growing *Lemna gibba* cultures to different nutritional conditions.

**Results:**

To establish a framework for quantitative metabolic research in duckweeds we derived a central carbon metabolism network model of *Lemna gibba* based on its draft genome. *Lemna gibba* fronds were grown with nitrate or glutamine as nitrogen source. The two conditions were compared by quantification of growth kinetics, metabolite levels, transcript abundance, as well as by ^13^C-metabolic flux analysis. While growing with glutamine, the fronds grew 1.4 times faster and accumulated more protein and less cell wall components compared to plants grown on nitrate. Characterization of photomixotrophic growth by ^13^C-metabolic flux analysis showed that, under both metabolic growth conditions, the Calvin-Benson-Bassham cycle and the oxidative pentose-phosphate pathway are highly active, creating a futile cycle with net ATP consumption. Depending on the nitrogen source, substantial reorganization of fluxes around the tricarboxylic acid cycle took place, leading to differential formation of the biosynthetic precursors of the Asp and Gln families of proteinogenic amino acids. Despite the substantial reorganization of fluxes around the tricarboxylic acid cycle, flux changes could largely not be associated with changes in transcripts.

**Conclusions:**

Through integrated analysis of growth rate, biomass composition, metabolite levels, and metabolic flux, we show that *Lemna gibba* is an excellent system for quantitative metabolic studies in plants. Our study showed that *Lemna gibba* adjusts to different nitrogen sources by reorganizing central metabolism. The observed disconnect between gene expression regulation and metabolism underscores the importance of metabolic flux analysis as a tool in such studies.

**Supplementary Information:**

The online version contains supplementary material available at 10.1186/s12870-023-04480-9.

## Background

The *Lemnaceae*, commonly known as duckweeds, are small, fast growing aquatic flowering plants. Prior to the emergence of Arabidopsis as a model organism, duckweeds were an important model system for plant biology [1]. *Lemnaceae* can be grown rapidly and axenically on defined media and have a high capacity for photomixotrophic or heterotrophic assimilation of various organic substrates [2–4] which makes them useful systems for isotopic tracer experiments in metabolic studies [5–7]. Given the asexual reproduction of duckweed fronds and their ability to grow on well-defined liquid growth media, growth rates and nutrient uptake rates can be measured with great precision, making them an excellent choice for metabolic research and metabolic modeling. The increasing availability of genetic resources should greatly benefit such efforts. In recent years, high quality genomes have been published for the duckweed *Spirodela polyrhiza* [8–12] and other *Lemnaceae* species (*Lemna gibba*, *Lemna minor, Wolffia australiana*) are currently in draft form [13, 14]. Furthermore, improved and highly efficient methods for stable genetic transformation and CRISPR/Cas9-mediated genome editing in duckweed species have been reported recently [15–17].

Nitrate and ammonium are often considered to be the two principal nitrogen sources for plants. Former studies reported a complex metabolic flux response to a change in nitrogen nutrition to cultured plant cells or tissues [18, 19]. While amino acids do not appear to be typical sources of nitrogen for plants growing in soil, it has been shown that developing plant embryos can be grown in culture with amino acids such as Gln or Asn as sole nitrogen sources [18, 20]. It could be argued that, while the bioavailability of amino acids in the root soil environment is limited, plants may be able to efficiently utilize organic nitrogen such as certain amino acids [21]. Therefore, it is of interest to compare the metabolic adaptation to nitrate versus that to an organic nitrogen compound as the sole nitrogen source in culture.

Here we present a model for the central metabolism of *Lemna gibba* clone 7742a (G3) based on a draft genome and transcriptomic sequencing data. We compared cultures of *L. gibba* growing photomixotrophically on glucose in liquid media under two metabolic growth conditions that differ in the nitrogen source (nitrate vs. Gln). Growth rate, carbon substrate consumption, biomass accumulation and biomass composition were quantitatively monitored under the two conditions. Targeted metabolic profiling, transcriptional profiling and differential gene expression analysis were performed in parallel. Based on feeding ^13^C-labeled glucose we applied ^13^C-Metabolic Flux Analysis (^13^C-MFA) to estimate the distribution of central metabolism flux under the two metabolic growth conditions. The model allowed us to distinguish patterns of carbon flow under the different growth conditions and to get insight into metabolic adaptations.

## Results

### Growth of *Lemna gibba* on different nitrogen sources

**Fig. 1:**
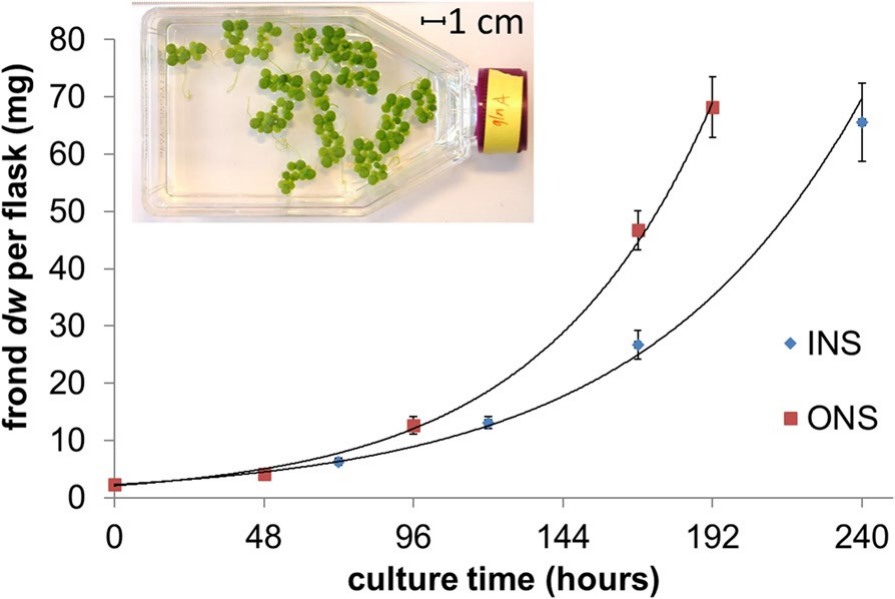
Growth kinetics of *Lemna gibba* cultured with inorganic nitrogen source or organic nitrogen source. *Lemna gibba* fronds were grown axenically under continuous light with glucose and nitrate (INS) or Gln (ONS) as nitrogen source. Growth was measured as frond area (Additional File 1: Table S1). The curve fits shown are exponential trend lines with R^2^ values above 0.99 (Microsoft Excel charts). The inset shows a photograph of a growing culture being used to measure surface area. Growth area was then converted into dry weight (*dw*) per flask based on the measured ratio of *dw* to fronds area (Table 1). Data points are mean ± SD (*n* = 3)

*Lemna gibba* was grown under continuous light and temperature on liquid media [22] with glucose as organic carbon source. Fronds were grown with two different sources of nitrogen, either with an inorganic nitrogen source (INS) as in the original Schenk & Hildebrandt medium recipe [22], or with an organic nitrogen source (ONS), in which case inorganic nitrogen was replaced with Gln to give the same total nitrogen concentration (13.7 mM). In the following, when describing and comparing experimental results, these two metabolic growth conditions are also referred to as INS or ONS condition. In order to record the growth dynamics under the two conditions, growth of cultures was monitored during 8 to 10 days (Fig. 1, Additional File 1: Table S1). Judging from the increase in frond area (Table 1), the initial inoculum was about 3% of the final *dw* harvested for both growth conditions, which is important for later ^13^C-labeling analysis since the inoculum is unlabeled. As demonstrated by the curve fits to frond *dw* (Fig. 1), cultures grew in an exponential fashion. The INS fronds grew at a rate of 0.014 ± 0.001 h^−1^ while under the ONS condition growth was 0.019 ± 0.001 h^−1^ (Table 1), which is 1.4-fold faster. Besides the differences in growth rate, a difference in frond density was observed. *L. gibba dw* biomass per cm^2^ fronds area was about 1.3-fold higher under the ONS growth condition (Table 1). Also, the relative proportion of root biomass was increased for the ONS condition (Table 1). Since the proportion of root dry matter in the total frond biomass was less than 10% (Table 1), roots and green matter were not analyzed separately. This means that all harvested frond biomass was analyzed.

**Table 1:** Growth parameters of *Lemna gibba* fronds cultured photomixotrophically on 100 mL culture medium with glucose (5 g/L) and different nitrogen substrates. Means and standard deviations represented three independent biological replicates

	INS	ONS
culture time (h)	240	192
Initial frond area (cm^2^)	1.1 ± 0.1	0.9 ± 0.1
Final frond area (cm^2^)	34.1 ± 3.5	27.2 ± 2.1*
Final dry weight (mg)	65.6 ± 3.5	68.1 ± 3.9
*dw*/frond area (mg/cm^2^)	1.9 ± 0.1	2.5 ± 0.1**
Fraction of DW in roots at harvest (% in *dw*)	2.79 ± 0.62	9.14 ± 2.66*
^13^C-enrichment of total dry biomass at harvest (%^13^C)^1^	21.1 ± 1.1	14.6 ± 0.1
doubling time (h)	50.5 ± 2.0	35.7 ± 1.0**
Specific growth rate (h^−1^)	0.014 ± 0.001	0.019 ± 0.001**

1, elemental analysis – isotope-ratio mass spectrometry after growing fronds with [U-^13^C_6_]glucose:glucose = 40:60 (mol/mol) (Additional File 1: Table S5)

*significant, *p* < 5%

**significant, *p* < 1%

### Effect of growth conditions on biomass composition

**Fig. 2:**
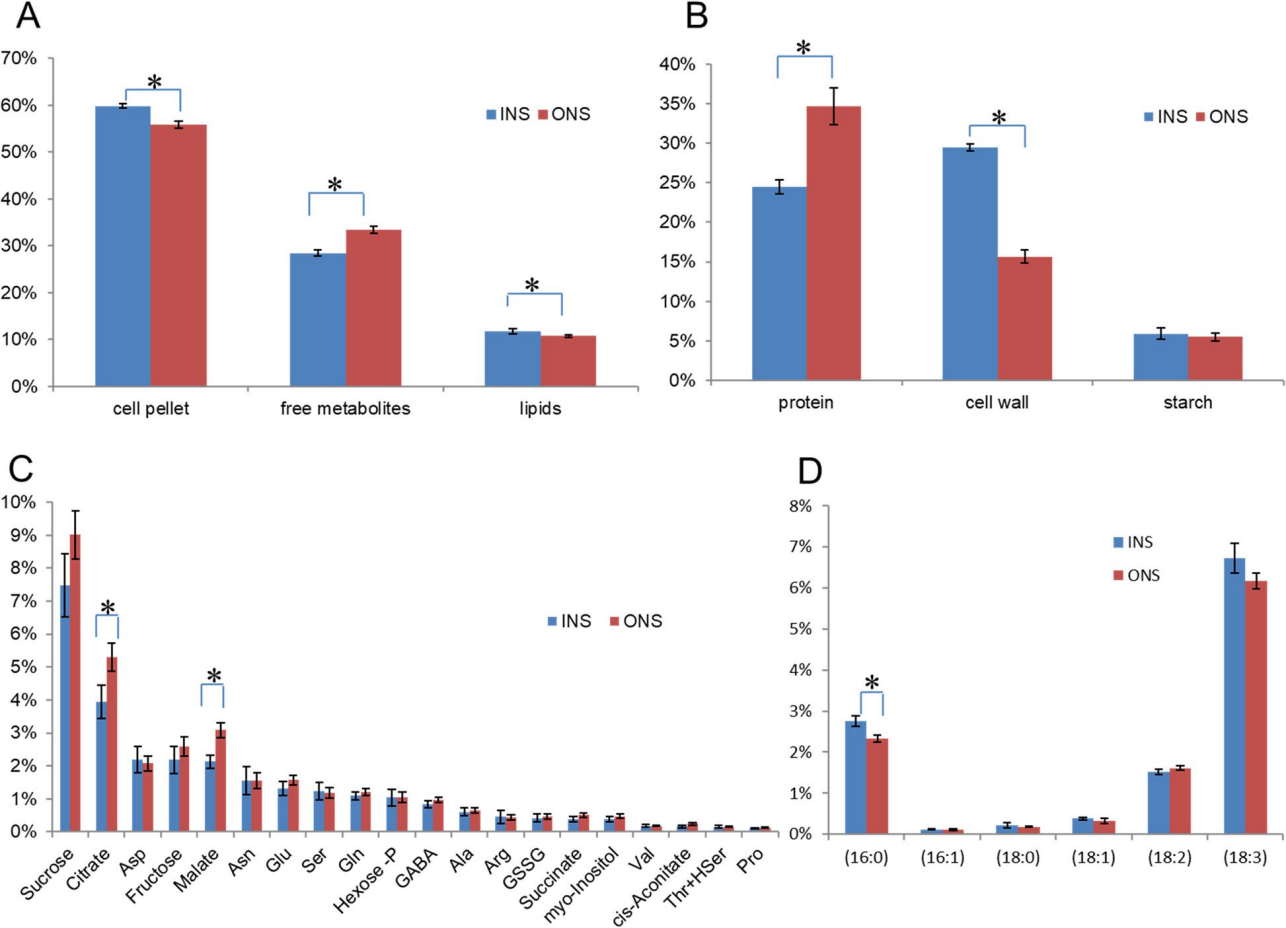
Biomass composition of *Lemna gibba* in percentage of total dry weight. **A** Biomass fractions of fronds. **B** Protein, cell wall polymer and starch content as derived by analysis of the cell pellet fraction. **C** Composition of the free metabolites fraction. To derive metabolic fluxes, only the 20 most abundant metabolites shown here were considered (more listed in Additional File 1: Table S3C). (D) Fatty acid composition of lipid fraction. Error bars indicate standard deviation (*n* = 3). All data are tabulated in Additional File 1: Table S3. *statistically significantly different from each other (*p* < 0.05)

The biomass of the harvested fronds was fractionated based on an organic biphasic solvent extraction procedure into a lipid fraction, a polar free metabolites fraction and insoluble cell material (cell pellet, Fig. 2A). Fronds cultured with ONS had slightly but significantly lower cell pellet and lipid content but were higher in free metabolites (Fig. 2A). More pronounced differences were obtained when the composition of the cell pellet fraction was analyzed (Fig. 2B). When cultured with ONS, fronds had a substantially higher protein content (40% more than cultured with INS medium) but only about half of cell wall fraction compared with cultured with INS medium (Fig. 2B).

The polar free metabolites fraction was analyzed by a targeted metabolic profiling approach, quantifying 57 metabolites (Additional File 1: Table S2). Among the 20 most abundant polar free metabolites, citrate and malate were significantly increased in the ONS condition compared to INS (Fig. 2C). The total increase in the free metabolites fraction seen under the ONS condition (Fig. 2A) can be mostly attributed to increases in sucrose, citrate and malate (Fig. 2C). In addition to the polar free metabolites, the lipid fraction was analyzed for its fatty acid composition. Consistent with previous reports [23] palmitic acid (16:0), linoleic acid (18:2, Δ9,12) and α-linolenic acid (18:3, Δ9,12,15) comprised approximately 90% of total fatty acids in *Lemna gibba* lipids (Fig. 2D). Relatively to the INS condition, culture with ONS slightly but significantly decreased palmitic acid (C16:0) (Fig. 2D).

### Uptake fluxes of organic substrates and fluxes into biomass

For both metabolic growth conditions, the glucose and Gln uptake rates were determined based on the reduction in medium glucose and Gln levels relative to dry matter formation (see Materials and Methods). We observed that glucose uptake was reduced under the ONS condition compared to the INS condition (Table 2), suggesting that Gln may serve as a source of reduced carbon in addition to glucose. Combining the biomass composition data (Additional File 1: Table S3) with the growth rates (Table 1), 22 fluxes into biomass were calculated (Table 3). Altogether, most biomass fluxes in the ONS condition are increased 1.3- to twofold relative to INS (Table 3), as expected from the observed increases in growth rate, free metabolite and protein levels in biomass (Fig. 2A, B). The flux of hexose phosphates into cell wall polymers is decreased under ONS (Table 3), consistent with the strong decrease in the cell wall fraction (Fig. 2B, vHPc_out).

**Table 2:** Rates of carbon uptake in growing fronds. Means and standard deviations represented three independent biological replicates

description	INS	ONS	Fold change (ONS/INS)
Glucose uptake (µmol⋅g -dw^−1^⋅h^−1^)^1^	102.3 ± 4.3	80.3 ± 2.5**	0.79
Gln uptake (µmol⋅g-dw^−1^⋅h^−1^)^1^	0	79.6 ± 2.1**	-
CO_2_ uptake (µmol⋅g-dw^−1^⋅h^−1^)^2^	589.7 ± 79.3	521.8 ± 56.4	0.88

1: Rates of uptake of glucose and Gln determined based on medium depletion. 2: Calculated based on vGlc_up, vGln_up and the average ^13^C-enrichments in of total dry biomass after growing fronds with [U-^13^C_6_]glucose:glucose = 40:60 (mol/mol) (Table 1)

**Significant (*p* < 1%)

**Table 3:** Fluxes into biomass of growing fronds. For definition of flux names see Additional File 1: Table S6. Means and standard deviations represent three independent biological replicates. Fluxes are given in absolute values. Unit: µmol⋅g-dw^−1^⋅h^−1^

Flux name	biosynthetic sink	INS	ONS	Fold change (ONS/INS)
vAlaP	Protein, free Ala	2.92 ± 0.18	4.85 ± 0.20**	1.66
vGluP	Protein (Glu, Pro, Arg), free Glu, Pro, GABA, citrulline, glutathione	6.53 ± 0.26	10.99 ± 0.36**	1.68
vGlnP	Protein (Gln), free Gln	3.00 ± 0.15	5.00 ± 0.17**	1.67
vSerP	Protein (Ser, Cys), free Ser	3.04 ± 0.32	4.49 ± 0.27**	1.48
vGlyP	Protein (Gly), free Gly, glutathione	1.99 ± 0.07	3.49 ± 0.15**	1.76
vHisP	Protein (His, Trp), free His, Trp	0.95 ± 0.03	1.65 ± 0.07**	1.74
VpheP	Protein (Phe), free Phe	2.26 ± 0.06	4.05 ± 0.18**	1.79
VtyrP	Protein (Tyr), free Tyr	1.76 ± 0.05	3.15 ± 0.14**	1.79
vValP	Protein (Val), free Val	2.28 ± 0.07	4.01 ± 0.16**	1.76
vLeuP	Protein (Leu), free Leu	2.93 ± 0.09	5.24 ± 0.22**	1.79
vIleP	Protein (Ile), free Ile	1.50 ± 0.04	2.67 ± 0.11**	1.78
vThrP	Protein (Thr, Met), free Thr, Met, homoserine	2.50 ± 0.08	4.43 ± 0.18**	1.77
vAsxP	Protein (Asp, Asn), free Asp, Asn	7.84 ± 0.64	11.88 ± 0.43**	1.52
vLysP	Protein (Lys), free Lys	2.20 ± 0.06	3.93 ± 0.17**	1.79
vGlyc_out	Lipid (glycerol moiety)	2.87 ± 0.05	3.36 ± 0.09**	1.17
vFASp	Lipid (fatty acids)	48.43 ± 0.87	56.67 ± 1.48**	1.17
vGlc_out	Hexose-phosphate, myo-inositol, sucrose	7.17 ± 0.66	10.90 ± 0.85**	1.52
vCit_out	Citrate, cis-aconitate	5.46 ± 0.57	8.20 ± 0.55**	1.50
vMal_out	Malate, succinate	4.45 ± 0.48	7.18 ± 0.55**	1.61
vFruc_out	Fructose	1.74 ± 0.35	2.65 ± 0.24**	1.52
vSt_out	Plastidic hexose phosphates into starch	5.24 ± 0.61	6.24 ± 0.60	1.19
vHPc_out	Cytosolic hexose phosphates into cell wall polymers	26.17 ± 0.50	17.76 ± 0.86**	0.68

^**^Significant (*p* < 1%)

### Carbon sources other than glucose and Gln contribute to biomass synthesis

**Fig. 3:**
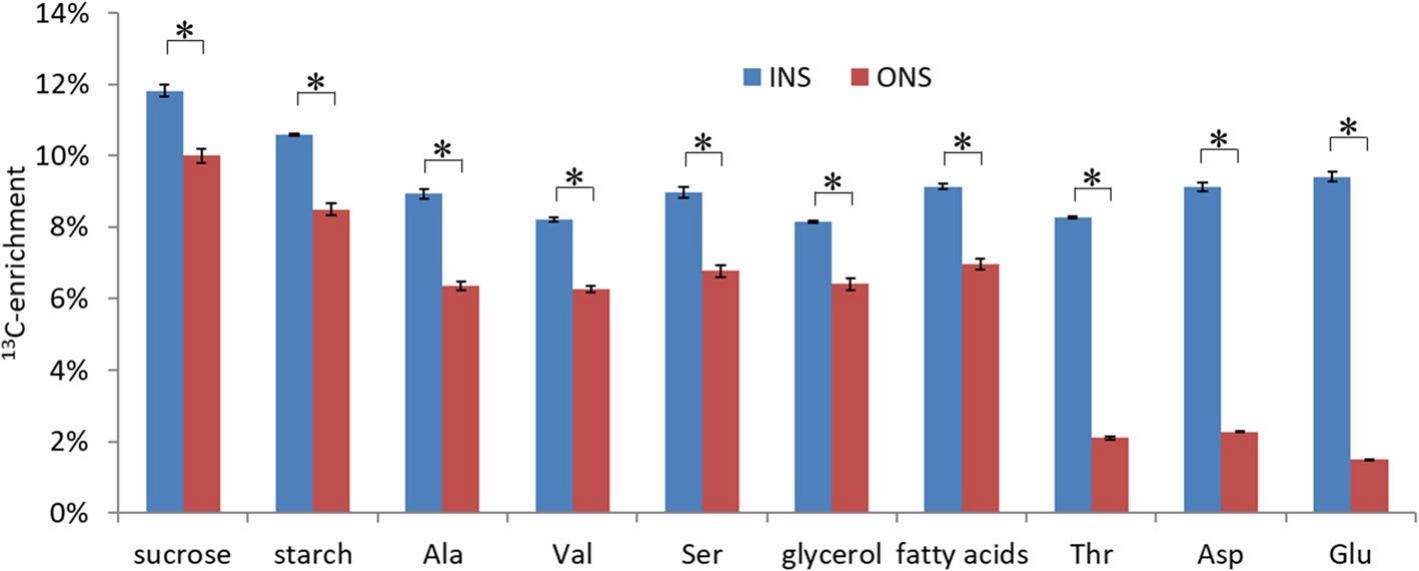
Incorporation of ^13^C-labeled medium glucose into various biomass components. Average^13^C-enrichment in biomass constituents according to mass isotopomer distribution determined by GC/MS (see also Additional File 1: Table S4A). The original mass isotopomer distribution was corrected for presence of 3.3% of unlabeled inoculate fronds biomass. Error bars indicate standard deviation (*n* = 3). *statistically significantly different from each other (*p* < 0.01)

For the purpose of analyzing metabolic flux using a central metabolism network, fronds were grown with a mixture of glucose, [1-^13^C]-glucose and [U-^13^C_6_]-glucose in a molar ratio 80:10:10 (See Materials and Methods). Mass isotopomer composition was determined by gas chromatography/mass spectrometry (GC/MS) in 16 analytes mostly derived from hydrolysis of biomass polymers (Additional File 1: Table S4B). Figure 3 shows the resulting average ^13^C-abundance for selected analytes. Under the ONS condition the contribution of unlabeled medium Gln is clearly evident since the ^13^C-enrichments are generally lower than under INS where no Gln is present (Fig. 3).

In particular, the amino acids Thr, Asp and Glu show substantial reductions in ^13^C enrichment (Fig. 3), as would be expected if Gln was deaminated to Glu and if Gln carbon was converted into Asp and Thr by passage through the tricarboxylic acid (TCA) cycle. Regarding the INS condition, the level of ^13^C-enrichment differs between analytes (Fig. 3), which is rather unexpected since labeled glucose is the only organic carbon source. If the shown biomass constituents would solely derive from glucose, they should be labeled close to 12.3%, the average ^13^C-enrichment in the medium substrate glucose. However, the ^13^C-enrichment of most of the analytes is substantially below that value (Fig. 3), suggesting that an unlabeled carbon source other than glucose contributes, such as atmospheric CO_2_. However, since glucose in our labeling experiment was labeled not equally at all carbon positions, the ^13^C content in biosynthetic products cannot be accurately predicted solely based on the average ^13^C-enrichment of glucose, which makes it somewhat uncertain if and how much atmospheric CO_2_ is involved.^1^ To determine the conversion of glucose-carbon to biomass more accurately, duckweeds were grown in another experiment with 40% of medium-glucose replaced by uniformly labeled [U-^13^C_6_]-glucose. The total frond biomass was then analyzed for ^13^C enrichment by elemental analysis – isotope-ratio mass spectrometry (Additional File 1: Table S5). While medium glucose was enriched with ^13^C at 40% and under the INS condition, biomass carbon was enriched at 21.1% (Table 1). This can only be explained by significant contributions from atmospheric, unlabeled CO_2_. Therefore, while the fronds grow with glucose as sole organic carbon source (Table 1), the CO_2_ fixation via the Calvin-Benson-Bassham (CBB) cycle seems to be very active as well. Based on the measured ^13^C enrichments in biomass (Table 1), calculation of the uptake rates of atmospheric unlabeled CO_2_ is possible (Table 2, See Material and Methods for calculations). Note that these CO_2_ uptake rates calculated from ^13^C enrichments in the INS and ONS case (Table 2) are not necessarily the net CO_2_ uptakes. CO_2_ can be taken up and released at the same time. In the ^13^C-MFA process discussed below both the uptake from the environment and the release of CO_2_ into the environment are included and determined.

### Definition of a *Lemna gibba* metabolic model

For the purpose of ^13^C-Metabolic Flux Analysis (^13^C-MFA) a carbon flux metabolic network of *L. gibba* central metabolism was defined based on genomic information and based on a generic plant carbon flux network (See Materials and Methods). An initial draft model of central carbon metabolism was constructed based on 21830 proteins predicted from a draft genome of *L. gibba* 7742a as part of the Lemna Genome Sequencing Project [13, 14]. Considering three subcellular compartments (cytosol, chloroplast, mitochondria), the network includes the glycolysis pathway, mitochondrial tricarboxylic acid (TCA) cycle, the oxidative pentose-phosphate pathway (OPPP), the Calvin-Benson-Bassham (CBB) cycle, the photorespiratory pathway and 22 lumped biosynthetic pathways to produce free metabolites, starch, protein, lipid and cell wall components. In the reconstruction process one potential pathway gap had to be dealt with. In a first network version, the oxidative steps of the OPPP were duplicated in the cytosolic and plastid compartment, while additional regenerative steps of the OPPP were only represented in the plastid compartment. This topology had a gap in that pentose phosphate, the product of the oxidative steps of the OPPP in the cytosol, cannot be further metabolized. Many higher plants feature a transport of pentose phosphates into the plastid, the xylulose 5-phosphate translocator (XPT). However, genes encoding XPT were not found in the *L. gibba* genome (see also Materials and Methods). Therefore, in order to render the cytosolic OPPP operable, the regenerative reactions of the pentose phosphate pathway were also duplicated into the cytosol. The final model structure is shown in Additional File 2, Fig. S2. Additional File 1: Table S6 lists 138 reactions and 303 associated *L. gibba* genes along with gene expression data in support of their activity (see Additional File 1: Table S7 for metabolites names). The reaction network was encoded as an input for the 13CFLUX2 flux modelling tool [24] (Additional File 1: Table S6, see model files in Additional File 3).

### Flux parameter fitting and model validation

**Fig. 4:**
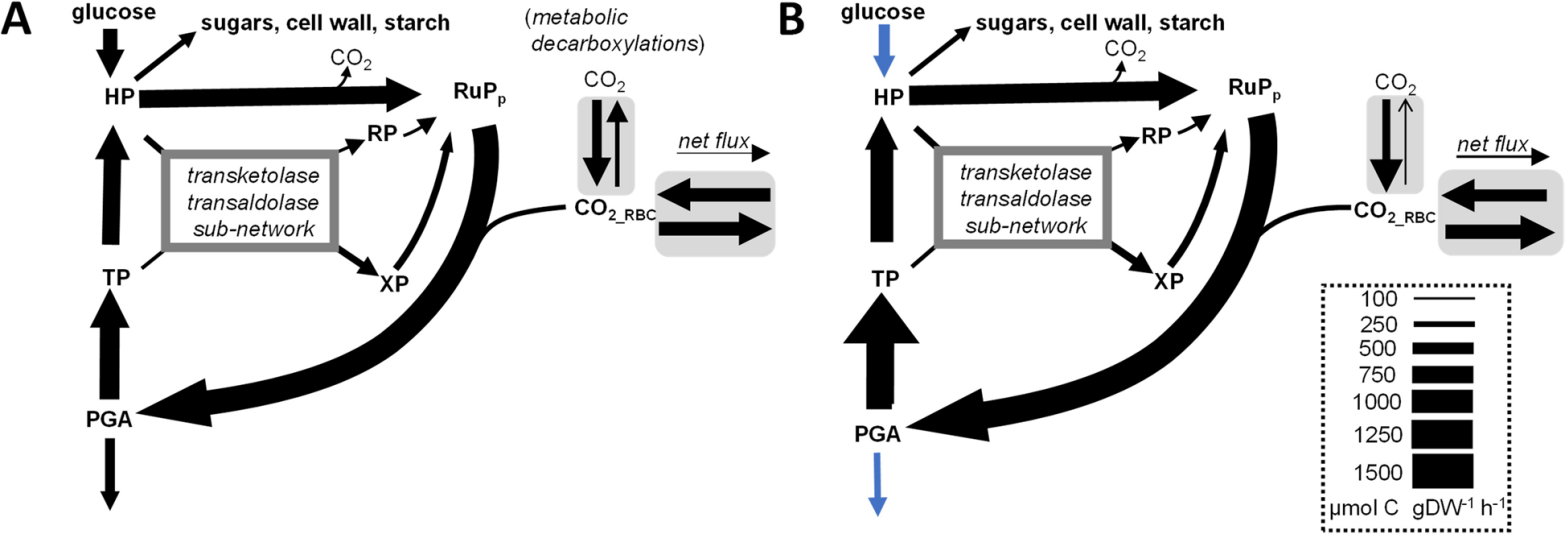
Flux maps for reactions associated to the Calvin-Benson-Bassham cycle in growing *Lemna gibba* fronds. Uncompartmentalized view of net carbon fluxes for the INS (**A**) and the ONS condition (**B**). Forward and reverse fluxes are highlighted in grey. Red / blue arrows: significantly higher / lower flux rate in ONS versus INS. See supplements for a more detailed flux map (Additional File 2: Fig. S3) and all flux values with statistical confidence measures (Additional File 1: Table S10C)

**Fig. 5:**
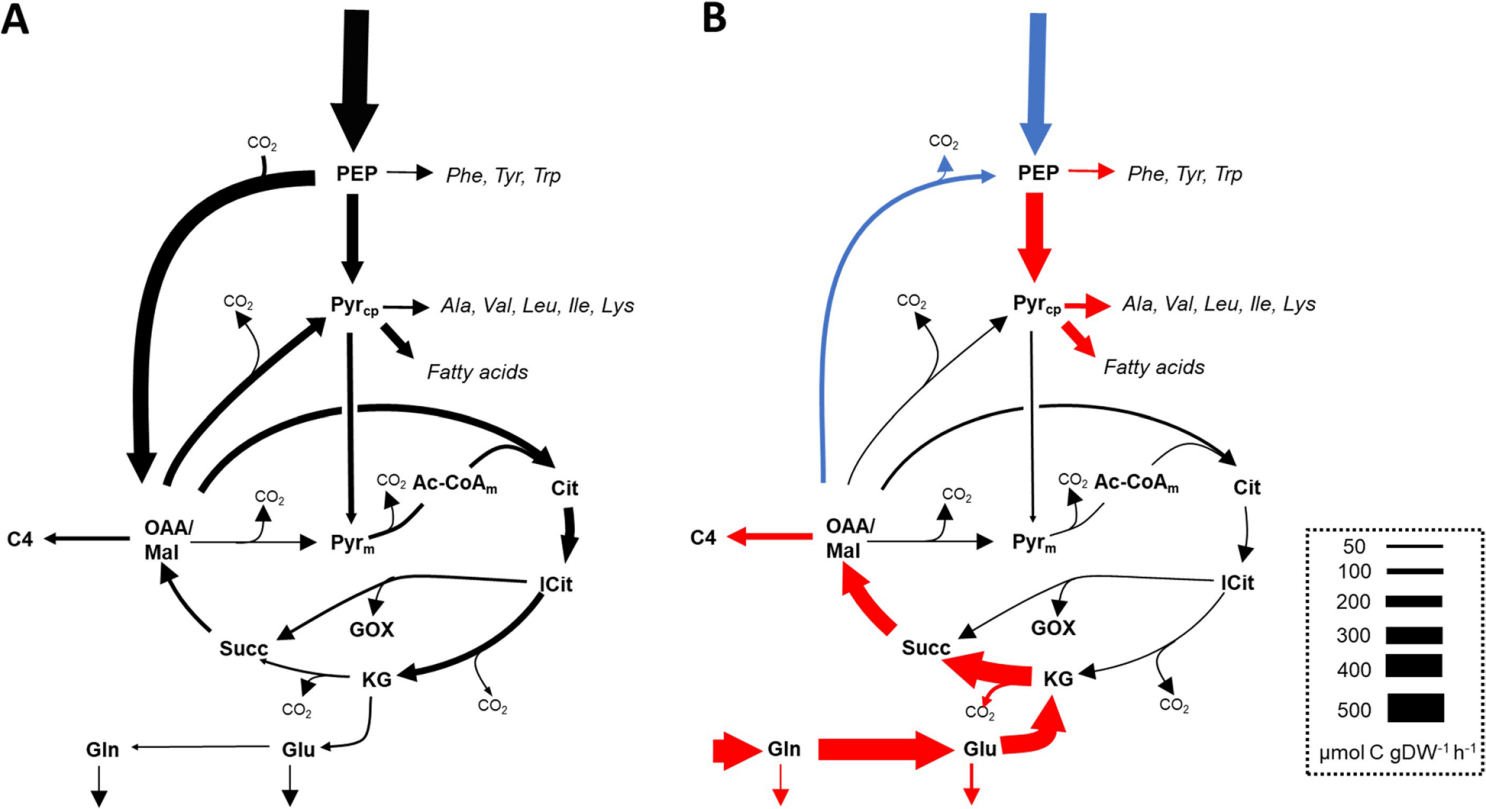
Flux map of reactions associated to the TCA cycle in growing *Lemna gibba* fronds. Uncompartmentalized view. Net carbon fluxes are visualized for the INS (**A**) and the ONS condition (**B**). Red / blue arrows: significantly higher / lower flux rate in ONS versus INS conditions. See supplements for a more detailed flux map (Additional File 2: Fig. S3) and all flux values with statistical confidence measures (Additional File 1: Table S10D)

The mass isotopomer composition in 27 molecular fragments in 16 analytes was used for iterative flux parameter fitting. One critical feature of the model structure relates to the uptake of atmospheric CO_2_ by the growing fronds as suggested by Fig. 3 and by an additional labeling experiment (Table 2). Three different model configurations were tested showing that enabling the uptake of unlabeled atmospheric CO_2_ is critical to obtain acceptable model fits (Additional File 1: Table S8; Additional File 2: Fig. S1; for further details see Material and Methods). An overview of the final network structure is given in Additional File 2, Fig. S2. All fluxes for the best fit solutions are listed in Additional File 1: Table S8. All net flux values are shown in a pathway map (Additional File 2: Fig. S3) and flux values are listed along with statistical confidence measures in Additional File 1: Table S10. For 49 out of 89 net fluxes, values differed significantly between the two growth conditions (Additional File 1: Table S10A, B). This includes most of the biomass fluxes listed in Table 3. Figures 4 and 5 show the flux results in an aggregated form, highlighting uncompartmentalized flux distributions associated to the CBB cycle and the TCA cycle, respectively. While in our metabolic network model glycolysis and OPPP occur in both the cytosol and the plastid, we find that the fluxes in this section are not well resolved in subcellular detail. The uncompartmentalized aggregated representation of the flux results (Fig. 4), on the other hand, has better statistical quality. This particularly applies to the oxidative steps of the OPPP (reactions vG6PDH_c_, vG6PDH_p_.). For each the INS and the ONS conditions, we could find optimum solutions for which either the cytosolic or the plastidic reaction become zero (Additional File 1: Table S9), showing that, based on the model fits to experimental data, it cannot be decided how the OPPP flux is distributed between the cytosolic or plastidic compartments. This finding is not surprising since other plant ^13^C-MFA studies have found that OPPP flux is very difficult to resolve particularly at subcellular resolution [25, 26]. In similar to the search for alternative optima, our statistical evaluation using Monte Carlo stochastic simulation (Materials and Methods) showed that particularly the flux through the plastidic reaction is poorly defined under both the INS and ONS conditions (vG6PDH_p_, Additional File 1: Table S10B). Yet, flux through the combined reactions shown in Fig. 4 is estimated to be 128.1 ± 50.4 and 141.4 ± 40.4 µmol⋅g -dw^−1^⋅h^−1^ under the INS and ONS condition, respectively (Additional File 1: Table S10C). In both cases, the standard deviations are less than 40% of the flux values, from which it can be concluded with good confidence that there is significant flux through the combined OPPP under both nitrogen nutrition conditions. Further details on the statistical evaluation of the OPPP flux values and additional support for this assessment based on flux confidence intervals determined by a parameter continuation strategy [27] are given in Additional File 2: Fig. S4.

### Under both metabolic growth conditions the Calvin-Benson-Bassham cycle is active together with the oxidative pentose phosphate pathway

Figure 4 shows that the CBB cycle is fully operational and active under both growth conditions. Most of the 3-phosphoglycerate (PGA) formed by the carboxylating function of RuBisCO is reduced to triose phosphate (TP), from which Ribulose 5-phosphate (RuP) is regenerated (Fig. 4). However, overall, a small net release of CO_2_ to the environment is found (Fig. 4). For both conditions, RuBisCO is re-fixing CO_2_ that is generated by decarboxylations elsewhere in metabolism (Fig. 4). In contrast to the canonical CBB cycle activity, a substantial part of the regeneration of RuP takes place via the CO_2_-generating oxidative section of the OPPP (Fig. 4). The ratio of decarboxylation by the OPPP to carboxylation by RuBisCO is 0.60 ± 0.13 and 0.60 ± 0.14 for the INS and ONS condition, respectively. This means that roughly 60% of the CO_2_ fixed by RuBisCO is released by the OPPP. Oxidative decarboxylation of hexose phosphate by the OPPP along with CO_2_ fixation and reduction via the CBB cycle, constitutes an energy (ATP) consuming metabolic cycle termed the glucose-6-phosphate shunt [28].

### Flux results are consistent with fast access of atmospheric CO_2_ to Ribulose-1:5-bisphosphate carboxylase/oxygenase

**Fig. 6:**
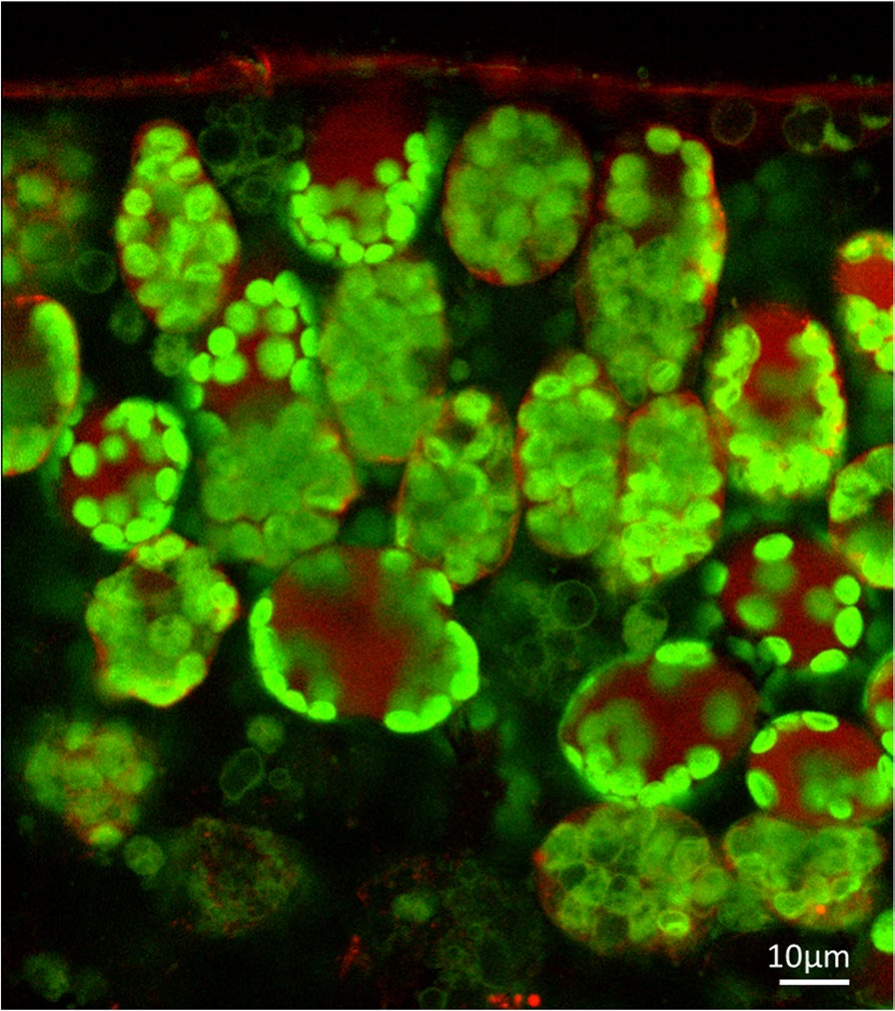
Localization of chloroplasts in mesophyll cells of growing *Lemna gibba* fronds by Confocal Laser Scanning Microscopy. Representative optical cross-section through the frond (6 cell layers of mesophyll are visible). Specific chlorophyll fluorescence visualizes the chloroplasts (color-coded green), while auto fluorescence of vacuole and cuticle is shown in red. This image is supported by the 3D-representation in Additional File 4: Movie

As seen in Fig. 4, the CBB cycle is highly active. Although no net CO_2_ fixation takes place (Fig. 4), model fits to the experimental labeling data were only satisfactory when uptake of atmospheric CO_2_ is allowed to take place simultaneously with CO_2_ release (See Materials and Methods). To explain this finding, one must be aware that duckweed fronds have a similar morphology to leaves of higher plants. In leaves of higher plants, the rapid diffusion of atmospheric CO_2_ across the intercellular air spaces to the cellular sites of RuBisCO activity is critical for maximal rates of photosynthesis [29]. Specific leaf morphological characteristics enable access of CO_2_ at high rates [29]. Similar to higher plants, when analyzing the cellular structure of growing *L. gibba* fronds by confocal laser scanning microscopy, the chloroplasts of mesophyll cells were found to be densely and tightly packed against the cell walls that face large intercellular air spaces (Fig. 6).

Since diffusion through liquid space is orders of magnitude slower than diffusion through air [29], this proximity of the chloroplasts to the airspace may allow rapid diffusion and exchange of CO_2_ between the intercellular airspaces and the chloroplast stroma, where RuBisCO is localized. In line with this idea, model fits indicated that a large fraction of the CO_2_ that is fixed by RuBisCO is derived from the atmosphere (Additional File 2: Fig. S1D).

### Flux associated to the tricarboxylic acid cycle differs substantially between the inorganic and organic nitrogen conditions

While the CBB cycle associated flux pattern shown in Fig. 4 does not differ substantially between the two metabolic growth conditions, there are two very distinct flux patterns associated with the TCA cycle (Fig. 5). Under the INS condition, carbon precursors for the Asp and Glu-related amino acids need to be produced *de-novo* from medium-derived glucose and inorganic nitrogen (Fig. 5A). To satisfy anabolic demands, PEP is carboxylated into oxaloacetate (OAA), which is a TCA cycle intermediate (Fig. 5A). α-Ketoglutarate (KG), the precursor of Glu and Gln is produced from OAA and acetyl-CoA via citrate and isocitrate (Fig. 5A). Under the ONS condition, not only is the carboxylation of PEP reduced, but the reaction flux is reversed (Fig. 5B). Flux reversal is possible by function of phosphenolpyruvate carboxykinase (EC 4.1.1.49), which we found to be expressed at comparable levels compared to phosphoenolpyruvate carboxylase (Additional File 1: Table S6). Gln uptake accounts for about 45% of total net carbon uptakes and some of the Gln C_5_ carbon chains are converted directly into Glu, Pro and Arg. Most Gln-derived carbon chains enter the TCA cycle (KG) and are further converted into malate and oxaloacetate, which are represented in the model as a merged pool (OAA/Mal) (Fig. 5B). OAA/Mal derived from Gln is then converted into Asp, Asn, Thr and Ile (C_4_, Fig. 5). Altogether, between the INS and the ONS condition, the TCA cycle operates to provide amino acids precursors in two distinct modes. In both cases the TCA cycle operates mostly in an anabolic mode. Of the total amount of carbon entering the TCA cycle, 84 ± 7% and 70 ± 23% leaves the cycle as organic acids, respectively.

### Differential expression and GO term enrichment analysis

In parallel to ^13^C-MFA, the two nitrogen conditions were compared by RNA sequencing (RNA-seq) to assess differences in expression of its 21,830 genes. Among 16340 genes with detectable transcription, 307 genes were found to be differentially expressed. Relative to the INS condition, 34 genes were found upregulated, and 273 genes were downregulated when cultured with ONS (Additional File 1: Table S11). Several transcripts with annotation as putative nitrate transporter as well as nitrite reductase and nitrate reductase transcripts were strongly downregulated under the ONS condition where nitrate is not available. To validate the differential expression data, the expression of Ferredoxin-nitrite reductase (EC 1.7.7.1, LEMGIv51_A5_006998_1), NADH-nitrate reductase (EC 1.7.1.1, LEMGIv51_A5_009812_1) and two additional enzymes (Transketolase, EC 2.2.1.1, LEMGIv51_A5_017459_1 and Pyruvate decarboxylase, EC 4.1.1.1, LEMGIv51_A5_020915_1) were analyzed by quantitative real-time reverse transcription PCR (qRT-PCR). The data show that the direction of change of transcript levels obtained by qRT-PCR were consistent with those obtained from the RNA-seq analysis (Additional File 2: Fig. S5). Significant changes in gene expression relevant to carbon-centric metabolic flux analysis are mapped to the reaction network in Additional File 1: Table S6. Seven of 311 mapped transcripts changed significantly between the conditions. For five of them, fluxes through associated reactions do not change between the INS and ONS condition (Additional File 1: Table S6). For example, the expression of one isoform of phosphogluconate dehydrogenase was found to be reduced under ONS, while the associated flux does not change significantly (Additional File 1: Table S6, LEMGIv51_A5_005055_1). There are three cases of coinciding significance calls. The expression of three isoforms of asparagine synthetase (glutamine-hydrolyzing, EC 6.3.5.4) was reduced under ONS (LEMGIv51_A5_015128_1, LEMGIv51_A5_015129_1, LEMGIv51_A5_015132_1; Additional File 1: Table S6). Asparagine synthetase forms Asn by transferring the amide group from Gln onto Asp, at the same time converting Gln into Glu. The enzyme is associated with reaction vGlnGlu, which summarizes interconversions between Gln and Glu that occur by asparagine synthetase and 10 other enzymes (Additional File 1: Table S6). Under the INS condition, a small net conversion from Glu to Gln takes place while flux direction is reversed under the ONS condition (Fig. 5). Since our model does not resolve the summarized enzyme reactions (vGlnGlu) in detail, it is unclear how exactly the downregulation of asparagine synthase can be explained in the network context. Apart from this it can be stated that, in *A. thaliana*, asparagine synthetase has been reported to be involved in the primary nitrogen assimilation [30]. The enzyme therefore might be nitrate induced, which may explain lower expression under the ONS condition.

The 307 differentially expressed genes (Additional File 1: Table S11) were further inspected by GO term enrichment analysis using the Database for Annotation, Visualization and Integrated Discovery (DAVID) web tool [31]. Since the DAVID web tool doesn’t recognize *L. gibba* as an organism, we first associated 20801 *L. gibba* protein sequences with Uniprot protein identifiers based on closest sequence homology (Additional File 1: Table S12). The full set of unique uniprot homologs was defined as the set of background genes for the GO enrichment analysis (See Materials and Methods). Based on a *p*-value cut-off of 5%, several of the obtained GO terms point towards redox processes, and in particular seven proteins annotated as class III secretable peroxidases that convert phenolic compounds into phenolic radicals (EC 1.11.1.7) were among them (Additional File 1: Table S13). Mapping the protein IDs back to *L. gibba* transcripts, 11 putative *L. gibba* phenolic peroxidases were found to be differentially expressed. 10 of these were downregulated under the ONS condition.

## Discussion

In this study, *Lemna gibba* was grown under low light conditions on liquid media supplemented with glucose as a carbon source. Growth and metabolism were compared for two different nitrogen sources, nitrate and Gln, by measuring growth kinetics and biomass composition, by metabolic profiling, by analyzing metabolic flux in central metabolism, as well as based on transcriptional profiling and differential gene expression analysis. The combined data gives insight into the metabolic adaptability of duckweeds.

The ^13^C-MFA showed that under both metabolic growth conditions the CBB cycle is fully operational while there is substantial OPPP flux as well (Fig. 4). In addition to the canonical CBB cycle, about half of RuP destined for RuBisCO is generated via oxidative decarboxylation of glucose-6-phosphate (the oxidative branch of the OPPP) (Fig. 4). Recently, this bypass of the CBB cycle via oxidative decarboxylation has been shown to occur in higher plants under photoautotrophic conditions (glucose 6-phosphate shunt) [28, 32–34]. The shunt releases one CO_2_ for every CO_2_ fixed, is balanced for NADPH but consumes 3 ATP for every CO_2_ turned around by oxidative decarboxylation and refixation, which constitutes a futile cycle [28]. For the OPPP to be active in the experiments of this study, the key enzymes of the pathway must be active in the light. Although it is often assumed that the activity of the OPPP is mostly suppressed in the light in order to avoid futile cycling, substantial activity of the glucose 6-phosphate shunt has been found under certain photosynthetic conditions in higher plants [28]. Although the glucose-6-phosphate shunt appears to be a futile (ATP-consuming) cycle, it likely has a function in stabilizing the CBB cycle or in adjusting the photosynthetic ATP/NADPH supply ratio to biosynthetic demands [28]. However, why the OPPP is highly active in *L. gibba* under the conditions used in this study is currently unclear.

Plant growth and biomass formation are strongly affected by both nitrogen availability and by its chemical form [35–37]. Although plants are considered to preferentially absorb and assimilate nitrogen as nitrate or ammonia, plants also have capacity to use organic forms of nitrogen [38–40]. For example, it has been shown that rice can be grown hydroponically with Gln as sole nitrogen source [40]. If *A. thaliana* is grown with ammonia and nitrate, addition of Gln promotes higher total biomass and relatively higher root biomass [39]. Consistent with this, if *Lemna gibba* fronds were grown with Gln as a nitrogen source, the rate of biomass formation and the fraction of the root biomass were increased relative to the inorganic (INS) condition (Table 1). One obvious explanation for faster growth on Gln is that use of this form of reduced nitrogen incurs substantially less energetic expense than the assimilation of nitrate [41]. This energetic advantage could also explain why significantly more protein accumulates under the ONS condition (Fig. 2B).

Nitrate has a dual role as both a key nutrient and a signal for nitrogen availability, inducing its own uptake and reduction [42–44]. Consistent with this, we found the expression of six genes encoding putative nitrate transporters as well as the expression of nitrate reductase and nitrite reductase to be strongly increased under the INS condition (Additional File 1: Table S11). However, other differences in flux distributions in central metabolism could not be related to changes in gene expression of the associated genes. The activity of sections of the TCA cycle and associated enzymes is known to be important for the provision of C skeletons and reductant for amino acid synthesis [45]. Our analysis revealed a substantial reorganization of fluxes around the TCA cycle, leading to differential formation of the biosynthetic precursors of the Asp and Gln families of proteinogenic amino acids depending on the nitrogen source (Fig. 5). However, due to the lack of correlation between changes in fluxes and gene expression, our data do not support the genetic regulation of these flux adaptations taking place. This observation parallels similar findings on the regulation of central metabolism made before in microbes and plants [18, 46–49] which emphasize the importance of metabolic regulation in central metabolism. For example, one enzyme known to be under complex (post-translational, allosteric) control is PEP carboxylase (PEPC) [50]. In addition, PEP carboxykinase (PEPCK) catalyzes the formation of PEP from oxaloacetate and is regulated by phosphorylation in plants [51]. We identified three genes for PEPC and three for PEPCK in the *L. gibba* genome. In our study, while PEP carboxylation took place under the INS condition, flux reversed to oxaloacetate decarboxylation and PEP formation under the ONS condition (Fig. 5B). Despite the flux reversal, expression of none of the genes encoding PEPC and PEPCK isoforms changed significantly (Additional File 1: Table S6). Regulation of enzyme activity could take place via protein phosphorylation. In a variety of plant studies the phosphorylation status of PEPC has been shown to be modulated by exogenous levels of sugars, phosphate or nitrogen sources [50]. In addition, plant PEPC is allosterically inhibited by malate, Glu and Asp [50]. In *L. gibba*, the level of malate was found to be higher under the ONS condition (Fig. 2C). The increased level in malate therefore is a likely cause of decreased flux through the PEPC reaction by allosteric inhibition of its activity.

The assimilation of nitrate and ammonia in higher plants is generally considered to take part mostly via the glutamine synthetase/glutamate synthase (GS/GOGAT) cycle [52, 53]. Gln is the initial product of ammonia assimilation via GS [53]. If Gln is the principal nitrogen source as in this study under the ONS condition, the GS/GOGAT cycle could likely be the entry point as well. However, neither the three putative GS genes nor the two putative GOGAT genes were expressed in *L. gibba* (LEMGIv51_A5_004150_1, LEMGIv51_A5_013274_1, LEMGIv51_A5_021303_1, LEMGIv51_A5_013691_1, LEMGIv51_A5_013918_1, Additional File 1: Table S11) Likewise, we were unable to identify other transcription responses that could be related to the induction of genes that specifically metabolize or transport Gln as a nitrogen source. For example, no differential expression was observed for 10 genes annotated as amino acid transporters. Nonetheless, at the level of metabolic flux, the entry of the Gln carbon chains into the TCA cycle was clearly evident (Fig. 5). This supports the notion that reorganization of flux to utilize Gln results from post-transcriptional mechanisms.

When nitrate is the major source of nitrogen, substantial amounts of ATP and NAD(P)H are needed to drive N assimilation [54]. Our *L. gibba* RNA-seq data show that one isoform of phosphogluconate dehydrogenase is expressed 32-fold higher under the INS condition relative to the ONS condition (LEMGIv51_A5_005055_1, Additional File 1: Table S11). Transketolase expression was higher under INS as well (LEMGIv51_A5_017459_1, Additional File 1: Table S11, Additional File 2: Fig. S5). This means that parts of the OPPP are upregulated under the INS condition. Similarly, in *A. thaliana* genes of the OPPP have been shown to be upregulated by nitrate [55]. However, in *L. gibba*, at the flux level no significant difference in OPPP flux was found between INS and ONS (Fig. 4). This finding provides further support for the importance of post-transcriptional mechanisms.

## Conclusions

Analyzing metabolite levels, transcripts and metabolic flux in parallel gave insight into how central metabolism in *L. gibba* adapts to grow with different nitrogen sources. Our results indicate that much of the metabolic adjustments taking place might rely predominantly on regulatory mechanisms at the metabolic level. With the experimental and analytical framework presented here we lay the groundwork for similar future studies on metabolic adaptability of duckweeds. The photomixotrophic condition we examined in this study is characterized by a simultaneous operation of the CBB cycle and the glucose 6-phosphate shunt. It would be interesting to assess how much cellular ATP is consumed by this futile cycling activity and how the overall energy cofactor balance is impacted. To further analyze the energy cofactor balance, a detailed stoichiometric model of *L. gibba* has to be derived, which then could be parametrized with physiological data and flux results from this study. Additional physiological measurements of photosynthetic activity, beyond the scope of this study, might also be required to better understand this condition.

Although we consider duckweed to be well suited for metabolic studies, cellular heterogeneity in multicellular organisms can have a confounding impact on flux estimation [56]. To further confirm the simultaneous operation of the glucose 6-phosphate shunt and the CBB cycle, effects of cellular heterogeneity within the growing fronds on the flux estimation process might be explored. Root cells, while constituting a small fraction of total fronds biomass (Table 1), will likely exhibit OPPP flux in absence of CBB cycle flux. Thus, in additional flux analysis experiments heterotrophic root tissue might be analyzed separately from the green photosynthetic tissues.

Duckweeds have potential for use as biofuel feedstocks due to their aquatic habit, rapid growth and capacity for carbon assimilation and storage [57–62]. Recently, accumulation of high levels of triacylglycerols in growing fronds of *Lemna japonica* was reported [63]. To further improve such lipid overproduction efforts, it might be important to study growth rate limitations due to high lipid synthesis rates. Here our study might be useful since we demonstrated how to address the relationship between metabolic growth conditions and achievable growth rates. To further explore duckweed as a biomass feedstock, it will be important to also explore metabolism under purely photoautotrophic conditions. This requires adaptations of flux analysis protocols for ^13^CO_2_ pulse labeling and Isotopically Nonstationary MFA (INST-MFA) [64]. The model we present in this study can be applied to INST-MFA studies with *L. gibba*. In addition, our results may help to refine current experimental INST-MFA protocols and models. For example, in various previous studies the labeling kinetics of some photosynthetic intermediates often cannot be fully understood in the context of the assumed model structure and modeling assumptions. In such cases, the existence of metabolically inactive pools was postulated or the involvement of large pools of slow-turnover neutral sugars in the carbon fixation process was considered and could explain the anomalies [33, 34, 65–67]. The existence of separate subcellular CO_2_ pools we established here by steady state ^13^C-MFA could be another alternative explanation for some of these phenomena. Thus, our insights might be helpful for improving INST-MFA models.

## Materials and methods

### Plant materials and growth conditions

Axenic cultures of *Lemna Gibba G3* clone 7742a (G3) were obtained from Rutgers University Duckweed Stock Cooperative ID DWC130) [68] and were cultured in SH medium (Sigma-Aldrich S6765, half as manufacturer’s recommended concentration, 1.6 g/L) [22] with 5 g/L glucose (pH 5.7). Fronds were cultured axenically in T-75 cell culture flask with vented caps (CytoOne®, CC7682-4875, USA Scientific, Ocala, FL) filled with 100 mL of the culture medium, at 24 °C, under continuous fluorescent light (100 µmol⋅m^−2^⋅s^−1^). The inorganic SH medium contains ammonium phosphate monobasic (1.3 mM) and potassium nitrate (12.4 mM) as nitrogen sources. For organic nitrogen growth medium these inorganic nitrogen containing salts were replaced by equal concentration of potassium phosphate monobasic and potassium chloride, respectively. Gln was added at 6.85 mM in order to provide nitrogen at the same concentration as in the SH medium. In general, to study the effect of the medium composition the fronds were acclimated to a new nutritional condition by pre-culturing for 10 days. Then a culture experiment was started with 2 to 3 fronds as inoculum. After 8 to 10 days of culture, fronds were harvested, and biomass composition was analyzed. To investigate the intracellular flux distribution, fronds were grown with medium glucose was replaced with glucose, [1-^13^C]-glucose and [U-^13^C_6_]-glucose (labeling at 99% ^13^C, Sigma-Aldrich) in a molar ratio 80:10:10. In another labeling experiment where ^13^C enrichment was determined in total biomass carbon, 40% of medium glucose was [U-^13^C_6_]-glucose (at 99%).

### Fronds surface area measurement and growth rate

Growth of cultures was quantified based on increase in frond surface area, using Scanalyzer BS (LemnaTec, Germany). The images were analyzed using LemnaLauncher image analysis software (LemnaTec, Germany) to obtain the total fronds surface area. Curve fits shown in Fig. 1 were generated with an exponential model using “trendline” in the chart generated by Microsoft Excel for Office365 (https://office.microsoft.com/excel). The growth rate was calculated by the following equation:

1 $$\upmu =\frac{{lnA}_{t}-{lnA}_{0}}{t},$$

where µ is the specific growth rate, *t* is culture time (*h*), *A*_*0*_ and *A*_*t*_ are the frond surface area at culture time 0 and *t*, respectively. The doubling time of duckweeds growth is $\frac{ln2}{\upmu }$.

### Substrate uptake measurements

To measure the uptake rates for medium glucose and Gln, growth experiments were repeated with unlabeled glucose. The initial and final medium glucose and Gln concentrations were measured to calculate glucose and Gln depletion and uptake rate. Glucose and Gln concentration were measured by mass spectrometry with internal isotope standards. To correct the water evaporation during the culture, a known amount of sorbitol was added to the medium after the culture. Briefly, after culture, 1 mL of sorbitol (0.7M) was added to a culture flask with originally 100 mL medium and mixed well. 100 µL medium and 1 mL water were then added to 100 µL of an aqueous solution of U^13^C_6_ glucose (30mM), U^13^C_6_ sorbitol (3mM) and D_5_-Gln (4.5mM). Then 2 mL CHCl_3_ was added, and the two phases were kept at 4°C for 20 min. After centrifuge at 3750 rpm for 20 min the upper phase was analyzed by direct infusion into a 4500 QTRAP mass spectrometry system (Sciex, MA, USA) in MRM mode. The mass spectrometry settings are listed in Additional File 1: Table S14. The metabolite concentrations were calculated based on the isotope ratios (unlabeled vs. isotope labeled standards). The exact medium volume was calculated based on the unlabeled sorbitol standard.

For exponentially growing cultures a substrate uptake flux can be calculated by multiplying the reciprocal of a yield coefficient with the specific growth rate. Accordingly, we define specific substrate uptake rates by:

2 $${V}_{uptake}=\frac{S-{S}_{0}}{D-{D}_{0}}\upmu$$

with *S*_*0*_ and *S* being the µmol amount of substrate in the culture vessel at the beginning and end of culture, respectively, and *D*_*0*_ and *D* the *dw* of fronds at the beginning and end of culture, respectively.

Besides the medium supplied organic substrates glucose and Gln, atmospheric CO_2_ is accessible to growing fronds as another carbon source. The CO_2_ uptake rate was determined based on the measured glucose and Gln uptake rates (above) and the fractional enrichment of ^13^C in biomass. For this purpose, fronds were grown with 40% of medium glucose replaced by [U-^13^C_6_]-glucose enriched at 99% ^13^C (average ^13^C-enrichment 40.3%). Dry biomass of harvested fronds was analyzed by elemental analysis – isotope-ratio mass spectrometry, resulting in measurements for ^13^C enrichment (*f*_*13C,BM*_). The analysis was performed at the University of California Davis Stable Isotope Facility (UCD SIF) using a PDZ Europa ANCA-GSL elemental analyzer interfaced to a PDZ Europa 20–20 isotope ratio mass spectrometer. Calculation of the CO_2_ uptake (*v*_*CO2_up*_) was done by the following relation. *f*_*13C*__*,BM*_ depends on all carbon-molar uptake fluxes and the ^13^C-enrichment in each of the carbon sources (40.3% in case of glucose):

3 $${f}_{13C,BM}=\frac{{6v}_{Glc\_up}\times 0.403+{5v}_{Gln\_up}\times 0.011+{v}_{CO2\_up}\times 0.011}{{6v}_{Glc\_up}+{5v}_{Gln\_up}+{v}_{CO2\_up}}$$

*v*_*GLC_up*_ and *v*_*Gln_up*_ are the uptake rates for glucose and Gln, respectively. Knowing *f*_*13C,BM*_, *v*_*GLC_up*_ and *v*_*Gln_up*_, *v*_*CO2_up*_ can be calculated after rearranging Eq. 3:

3a $${v}_{CO2\_up}=\frac{{6v}_{Glc\_up}\left(0.403-{f}_{13C,BM}\right)+{5v}_{Gln\_up}\left(0.011-{f}_{13C,BM}\right)}{{f}_{13C,BM}-0.011}$$

Note that *v*_*CO2_up*_ is the uptake rate of unlabeled atmospheric CO_2_, which is not identical to the net CO_2_ balance since cellular CO_2_ can also be released. To estimate the statistical uncertainty in *v*_*CO2_up*_, Eq. 3 was applied repeatedly after pseudo-randomization (re-sampling) of values for *f*_*13C,BM*_, *v*_*GLC_up*_ and *v*_*Gln_up*_ according to their mean and standard deviations [69].

### Biomass composition analysis

Cultures were kept in tissue culture flasks with vented seal caps. Cultures grew just until the fronds covered the entire culture flask surface area to ensure they were under exponential growth stage. Fronds were harvested by filtration, extracted and dry weight fractions of cell pellet, lipids and free metabolites content were determined as described before in detail [25]. Briefly, tissues were first ground in 3 mL methanol/water (4:3, v/v) with an Omni tissue grinder followed by adding 3.4 mL CHCl_3_ to result in a biphasic solvent system (CHCl_3_/methanol/H_2_O, 8:4:3, v/v/v) [70]. The biomass fractions of lipids (CHCl_3_ phase), free metabolites (methanol/water phase) and cell pellets (insoluble material) were obtained by weighing dry weight of each phase. The total nitrogen amount in cell pellets was measured by CHN elemental analysis (vario MICRO cube, ELEMENTAR Analysensysteme GmbH, Germany). The nitrogen was then converted to protein weight based on the amino acid composition of duckweed protein [71].

### Starch content assay

Starch content was measured by modified protocol [72]. Briefly, harvested plant material was filtered to remove medium and transferred to a tube containing 5 mL 80% ethanol and incubated in a boiling water bath for 3 min. The samples were centrifuged, the supernatant discarded, and the same ethanol extraction repeated twice. The dry pellet was homogenized with 5 mL water and 1 mL homogenate was heated to 100°C for 1 h. 0.1 mL 50 mM acetic acid solution (pH 4.8, adjusted by 10 M NaOH) containing 14 U/mL amyloglucosidase and 63 U/mL alpha-amylase was added to 1 mL homogenate and starch was digested at 55°C for 1 h. After centrifugation, glucose concentration was measured in the supernatant by glucose oxidase reagent (Thermo Scientific, TR15221).

### Fatty acid profiling

The lipid fraction was derivatized by boron trichloride methanol to fatty acid methyl esters (FAMEs) [73]. Fatty acid profile was obtained by GC–MS (6890N gas chromatograph with 5973 quadrupole mass spectrometer; Agilent Technologies) with a J&W DB 23 capillary column (30 m × 0.25 mm × 0.25 mm) by using scan mode. The injector was held at 250°C and the oven temperature was heated from 80°C to 170°C at 20°C⋅min^−1^ and from 170°C to 210°C at 5°C⋅min^−1^. To quantify the concentration of fatty acids, 100 µg heptadecanoic acid (17:0) was added prior to transmethylation to each sample as an internal standard. Quantification was done by peak area comparison with the internal standard using MSD Chemstation Data Analysis software to integrate GC–MS peak areas (total ion current).

### Free metabolites profiling

The free metabolites fraction from the biphasic CHCl_3_/methanol/H_2_O fractionation of frond biomass was analyzed by Hydrophilic interaction chromatography (HILIC), using an aminopropyl column (Luna NH_2_, 250 mm × 2 mm, particle size 5 µm, Phenomenex, Torrance, CA) according to a protocol by Bajad et al*.* (2006) [74]. A liquid chromatography system (Ultimate 3000, Dionex, Idstein, Germany) was coupled to a triple quadrupole mass spectrometer (ABSciex, Darmstadt, Germany). To obtain a maximum of different compounds the column was used in combination with the mass spectrometer in negative and positive mode and varying eluent gradients. Mass spectrometer settings for 70 analyzed metabolites are listed in (Additional File 1: Table S15). The LC eluents are: Solvent A, acetonitrile; Solvent B: 20 mM ammonium acetate + 20 mM ammonium hydroxide in 95:5 water:acetonitrile, pH 9.45. 40 metabolites were determined in negative mode after separation with the following gradient: t = 0, 25% B; t = 8 min, 30% B; t = 22 min, 100% B; t = 32 min, 100% B; t = 33.5 min, 25% B; t = 44 min, 25% B. The gradients in the positive mode to determine 30 additional metabolites are as follows: t = 0, 20% B; t = 6 min, 25% B; t = 14 min, 100% B; t = 24 min, 100% B; t = 25 min, 20% B; t = 30 min, 20% B. The quantification was done by external calibration (4 concentrations, 3 technical replicates each). 57 metabolites were quantified using authenticated chemical standards (Additional File 1: Table S15). 30 of these, accounting for about 90% of the weight of the measured metabolites, were included into the flux modeling process (Additional File 1: Table S3C).

### Determination of biomass fluxes

Fluxes from central metabolism into cellular biomass components (biomass fluxes) were calculated based on the biomass proportions of lipid, protein, starch, the cell wall fraction and 30 most abundant free metabolites. The weight of the polymers was divided according to their respective monomer composition. For this, the protein composition was estimated by averaging 10 literature reports on the amino acid composition in duckweed [71]. The weight of the lipid fraction was accounted for as triacylglycerol with the measured fatty acid composition. The weight of the cell wall fraction was accounted for as a glucose polymer. To obtain flux values the molar abundances of biomass compounds were multiplied with the specific growth rate of the growing fronds. The statistical uncertainty in the biomass fluxes was derived by adding random noise to the biomass composition data according to their standard deviations. Using the random number generator function in Microsoft EXCEL, normal distributed numbers were derived according to mean and standard deviation [69]. Standard deviations in the biomass derived fluxes were then derived based on 20 times repetition of this procedure.

### ^13^C isotopomer measurement

The ^13^C labeling pattern of various metabolites was measured by GC–MS (6890N GC/5975 quadrupole mass spectrometer; Agilent Technologies) with select ion monitor (SIM) mode as previously reported [25]. All analyses were in splitless mode, with 1 μL injection volume, and the carrier gas was helium at a flow rate of 1 mL⋅min^−1^ with an HP-5MS column (30 m × 0.25 µm × 0.25 µm, Agilent). Amino acids were derivatized by N-methyl-N-(tert-butyldimethylsilyl) trifluoroacetamide (MTBSTFA) [75], the injector temperature was set to 275°C and the column temperature was programmed as follows: initial, 100°C, 4 min; to 200°C at 5°C⋅min^−1^; to 300°C at 10°C⋅min^−1^; final time, 5 min. Glucose from digested starch was converted to methyl ester alditol acetates (MAAs) as previously reported [76]. The injector temperature was 250°C and the column temperature was programmed as follows: initial, 100°C, 4 min; to 300°C at 20°C⋅min^−1^; final time, 2 min. In order to analyze sucrose the free metabolites fraction was derivatized by methoxyamine-HCl and followed by N-Methyl-N-(trimethylsilyl) trifluoroacetamide (MSTFA) [77]. Injector temperature was set to 250°C; initial, 70°C, 4 min; to 310°C at 5°C⋅min^−1^; final time, 10 min. Fatty acid methyl esters (FAMEs) were further reduced to saturated fatty acid methyl esters by PtO_2_ under H_2_ pressure and only the carboxyl group carbon (e.g. C1 and C2) labeling information were measured. To suppress the contaminant fragments around base peak of fatty acid methyl esters (m/z 74), the ionization energy for quadrupole mass analyzer was reduced from the standard 70eV to 15eV [25], the injector temperature was 250°C and the column temperature was programmed as follows: initial, 90°C, 4 min; to 240°C at 10°C⋅min^−1^; final time, 15 min. Glycerol was converted to trifluoroacetic acid ester by trifluoroacetic acid anhydride (TFAA) as previous report [78], the injector temperature was 250°C and the column temperature was programmed as follows: initial, 60°C, 4 min; to 110°C at 10°C⋅min^−1^; to 250°C at 20°C⋅min^−1^; final time 1 min. Altogether, 29 selected fragments in 17 analytes were monitored using the selected ion monitoring mode of the mass spectrometer. Correction for naturally occurring isotopes (C, H, N, O, S, Si) in the derivative side chains was applied as reported before [25]. In total 120 MS measurements were averaged from three independent labeling experiments. The resulting standard deviations of MS measurements were adjusted so that all values were at least 1% on the fractional enrichment scale. This procedure makes it easier to pass the statistical test for goodness of fit at the expense of increased uncertainty in flux parameter confidence intervals [79].

### Whole transcriptome sequencing and data processing

Fronds grown on inorganic nitrogen or on organic nitrogen medium were harvested and immediately frozen in liquid nitrogen. For each condition three biological replicates were analyzed. Total RNA was extracted by grinding 200 mg frozen tissue to a fine powder in liquid nitrogen with a mortar and pestle, followed by extraction with TRIzol Reagent (Invitrogen, MA, USA) and ethanol precipitation. Total RNA was resuspended and treated with RQ1 RNase-free DNase (Promega,WI, USA) on an RNA Clean & Concentrator-5 column (Zymo, CA, USA). The purity and concentration of RNA were determined by a NanoDrop Nd-1000 spectrophotometer (Thermo Scientific, MA, USA) and a Qubit fluorometer (Qiagen, MD, USA). Polyadenylated RNA was enriched from total RNA with the Dynabeads mRNA Purification Kit (Life Technologies, CA, USA). Indexed, directional RNA-seq libraries were prepared with ScriptSeq v2 reagents (Epicentre, WI, USA) from 40–50 ng of polyA-selected RNA according to the manufacturer’s instructions. Libraries were sequenced on an Illumina HiSeq 2000 instrument (Illumina, Inc., CA, USA) generating 101 bp paired-end reads. Reads from each sample were mapped against the 21,830 predicted transcripts of the *Lemna gibba* draft genome v0.5.1 [80] using Bowtie [81].

### Differential expression analysis

Differential expression analysis for the two conditions (INS, ONS) was performed using the DESeq package [82, 83]. This procedure included normalization of RNA-seq read count data (estimateSizeFactors, using default parameters) and the negative binominal test as implemented in DESeq. Significant differential expressed genes were defined for an adjusted *p*-value ≤ 5% (Benjamini–Hochberg adjustment for multiple testing) and a more than four-fold change in gene expression between conditions.

### Quantitative reverse transcription real-time PCR (qRT-PCR)

A few cases of significant differentially expressed genes were validated by quantitative PCR. Total RNA from samples was isolated using TRIZOL reagent (Invitrogen, MA, USA) as described in the manufacturer's user manual. First-strand cDNA was synthesized by using cDNA synthesis kits from Fermentas, (ThermoFisher, MA, USA). Two reference housekeeping genes from Arabidopsis (F-BOX AT5G15710, and Expressed1 AT4G33380) were chosen from literature [55, 84] and the corresponded *Lemna gibba* genes were identified by BLAST search against the predicted transcripts. The primers for real-time PCR were designed and synthesized by Integrated DNA Technologies (Coralville, IA, USA). The sequences of primers for reference and target gene are listed in (Additional File 1: Table S16). Real-time PCR was performed in 96-well plates with a Bio-Rad CFX96 Touch Real-time PCR Detection System. Reactions were in 10- µL volumes containing 2 µL cDNA (0.5 µg/µL), 0.3 µL each primer (10 mM), 2.4 µL water and 5 µL Bio-Rad iQ™ SYBR® Green Supermix (Bio-Rad, CA, USA). Data were analyzed by CFX Manager™ Software (Bio-Rad, CA, USA).

### GO term enrichment analysis

Analysis of Gene Ontology (GO) terms which are enrichment in differentially expressed genes was performed with the online bioinformatics resources given by the Database for Annotation, Visualization and Integrated Discovery (DAVID) (v6.8) [31, 85]. To use GO term enrichment with this tool, the genome-predicted gibba protein sequences were mapped onto their closest homologs in the UniProtKB/Swiss-Prot protein database (file ‘uniprot_sprot.fasta’ with 553,655 protein sequences accessed on February 15, 2017 at ftp://ftp.uniprot.org) [86] using Protein–Protein alignment (BLAST ver. 2.2.28 +) [87]. Top hits with e-value ≤ 10^–20^ were kept, which associates 12,673 of the 20801 *L. gibba* protein sequences with 8,597 unique Uniprot sequence identifiers (Additional File 1: Table S12). These defined the genomic background in the DAVID online resource. Of the 307 L. gibba genes found to be significant differentially expressed 218 were associated to 179 unique Uniprot IDs, for which enrichment of GO terms was assessed against the genomic background. The GO term categories Biological Process, Cellular Component, Molecular Function, KEGG pathway and EC Number were analyzed. A *p*-value cut-off of 0.05 was chosen (Benjamini–Hochberg adjustment for multiple testing).

### Construction of *Lemna gibba* metabolic network

A metabolic network was derived from a *Lemna gibba* v0.5.1 draft genome [13, 14] by using the Ensemble Enzyme Prediction Pipeline (E2P2, version 3.0) [88], an enzyme annotation pipeline that has been used by others to generate metabolic pathway databases from plant genomes for Plant Metabolic Network [89]. 21830 protein sequences predicted for 21830 *L. gibba* gene loci were given as input, returning a total of 6875 proteins with a predicted enzyme function. 4436 of these are enzyme functions acting on small molecules. A network of central carbon metabolism in *Lemna gibba* to be used for ^13^C-metabolic flux analysis was then defined by mapping the E2P2-predicted reactions onto a carbon flux network originally developed for *Brassica napus* [90]. Plant central metabolism is of large complexity. Due to limitations in computational performance, the network topology regularly needs tailoring of the detailed topology to a smaller size by lumping reactions or merging metabolic pools across subcellular compartments. The key design considerations used to define a lumped ^13^C-MFA network for *B. napus* have been discussed elsewhere [91]. Most of these model simplifications have been retained, so that the *B. napus* model can be understood here as a generic scaffold for the central metabolism of higher plants. A manual curation process was applied to improve the reaction-gene associations. If multiple isoforms of an enzyme were identified by E2P2, the likely subcellular localization could often be determined based on finding the closest *Arabidopsis* homologs in the reference model by bidirectional BLAST searches (*L. gibba* protein sequences against Arabidopsis TAIR10 predicted proteins and vice versa). In many cases the subcellular localization of *L. gibba* protein sequences was predicted using the online tool TargetP [92]. In addition to the enzyme functions predicted by E2P2, a number of key transmembrane metabolite transport functions were predicted based on transporter functions from the reference model. As particularly relevant to central metabolism we identified members of a family of phosphate translocators that can exchange various three to 5-carbon phosphorylated intermediates of glycolysis and the pentose-phosphate pathway (triose phosphate, PEP, xylulose-5-phosphate, glucose-6-phosphate) between the cytosol and chloroplast compartments. Four functional subclasses of the plastid phosphate translocators are known in Arabidopsis [93] and phylogenetic analysis of protein sequences of these subclasses among higher plant species has indicated that the four cluster apart [94]. Searching the genomes of *L. gibba* and *Spirodela polyrhiza* we could identify gene homologs for all four subclasses, except genes encoding the xylulose 5-phosphate translocator (XPT). Based on phylogenetic analysis of the phosphate translocator family across about 100 sequenced plant genomes it was found that a loss of the XPT occurred within subphyla of the monocots [94]. It therefore seems likely that the monocot group of duckweeds lost the XPT as well. The XPT has been suggested to be an important link between the pentose-phosphate cycles (interconverting triose-, pentose- and hexose phosphates) in the cytosol and the plastid [93, 95]. If the XPT is missing then the cytosolic OPPP cannot operate unless the enzymes of the pentose-phosphate cycle (transaldolase, transketolase, ribulose-phosphate 3-epimerase, ribose-5-phosphate isomerase) are present in the cytosol to convert pentose-phosphate into hexose phosphate. While we could identify genes encoding the latter enzyme activities in the *L. gibba* genome, our analysis of the genetic data left open whether enzyme isoforms exist for both the cytosol and the plastid. Despite this, the model was designed with complete cycles in both compartments in order to allow the OPPP to work in both compartments in absence of XPT. In light of these uncertainties in network definition, the interpretation of fluxes through OPPP and glycolysis at subcellular resolution must be done with caution. Nevertheless, we kept the compartmentalized scaffold. An aggregated uncompartmentalized view can always be generated and inspected. Any flux solution with statistical measures of uncertainty attached ​​can easily be projected onto a non-compartmentalized or otherwise lumped network version, based on linear combinations of fluxes (e.g. by adding the cytosolic and the plastidic pyruvate kinase flux, for example). Flux ratios can be generated in the same way. When using a Monte Carlo approach to generate the statistics (repeated iterative flux parameter fitting with noise-added measurement data), the aggregation of the best fitting flux solution and the flux solution vectors representing the statistical uncertainty is done in the same way. This means that besides the summation of corresponding flux values, no special statistical procedure needs to be developed to obtain flux statistics for the aggregated network.

Regarding the activities of ribulose-1,5-bisphosphate carboxylase/oxygenase (RuBisCO, EC 4.1.1.39), the ratio of carboxylation to oxygenation is largely dependent on the concentrations of both substrates present at the site of RuBisCO activity. Under normal atmospheric conditions, about 25% of the reaction catalyzed by RuBisCO is oxygenation [96]. It has been reported for the duckweed *Spirodela polyrhiza* that under photomixotrophy, when organic carbon substrates are available and may be catabolized, RuBisCO oxygenation and operation of the photorespiratory pathway are most likely largely suppressed due to elevated intracellular CO_2_ levels [3]. Therefore, in our model simulations, photorespiration was suppressed by setting the ratio of carboxylation flux to oxygenation flux to 1000:1.

### Flux estimation

Here we apply steady state ^13^C-MFA, i.e., isotope tracer-based metabolic flux analysis under metabolic and isotopic steady state [91]. Supporting these assumptions is that the cultures were grown under continuous light. Also, based on the experimental determination of substrate uptake rates, less than 20% of medium glucose was depleted under both conditions and about 40% of the medium Gln was consumed under ONS. Since exponential growth is maintained (Fig. 1), we can assume that none of the nutrient supplies becomes limiting and that the fronds grow and divide at their maximal rate possible under the INS and ONS conditions, respectively. During the growth in culture the frond biomass increased about 30-fold under both conditions (Table 1) which means that the fronds steadily divided about 4 to 5 times. We conclude that the metabolic and isotopic steady state assumption in ^13^C-Metabolic Flux Analysis [97] is sufficiently approximated. ^13^C-Metabolic flux analysis was mostly performed with the software tool 13CFLUX2 [24]. Parts of the analysis was also done with influx_s version 6.1 [98]. General methodology can be found in [99]. In order to avoid local optima, model optimization (minimisation of the sum of square of the difference between the measured and simulated labeling profile) was repeated at least 100 times for each model, each time with randomized start values for the free fluxes. The IPOPT optimization algorithm [100] was selected with the maximum number of iterations before termination set to 2000. To test if a flux solution is statistically acceptable the theoretical χ^2^ value was calculated based on the general model information. Degree of freedom is the number of mass isotopomer measurements minus the number of measurement groups, plus the number of measured flux rates minus the number of free net and exchange fluxes in the model (Additional File 1: Table S8).

### Testing of different model configurations

Regarding the uptake of atmospheric CO_2_ three model configurations were tested. For the first one, only net CO_2_ efflux is present so that no uptake of atmospheric CO_2_ is possible (Additional File 2: Fig. S1A). In this case, for the INS model, the goodness-of-fit measure between experimental labeling signatures and model simulation was 368 (sum of squared residuals, SSR), which is about 6 times higher than acceptable by the statistical test of goodness of fit (χ^2^ = 63 based on a 90% confidence level, Additional File 1: Table S8). Next, a new CO_2_ pool was added to the model to be used as substrate by RuBisCO (“CO2_RBC”). This pool can exchange CO_2_ with atmospheric CO_2_ by independent uptake and release reactions while it can also exchange with the common cellular CO_2_ pool that is used by carboxylase and decarboxylase reactions other than RuBisCO (Additional File 2: Fig. S1B). In the model iterative fitting process, the extent of exchange between the two pools is freely adjustable, allowing for flux solutions where the two are poorly mixed as well as solutions in which they are quasi fused and in complete isotopic equilibrium. This modification resulted in a dramatic improvement in the fit of the INS model, with both the INS and the ONS models passing the statistical goodness-of-fit test (Additional File 2: Fig. S1B). However, it was found that based on iterative fitting of the model to isotopomer data particularly the input and efflux of CO_2_ are weakly determined. Beneficial here was the inclusion of uptake rates for medium glucose, Gln and CO_2_ as determined from additional experiments (Table 2). Adding these uptakes to the model as flux measurements led to improvements in the statistical confidence measures of the fluxes. Under this model configuration (Additional File 2: Fig. S1C) and for repeated optimization with randomized initial values for free fluxes, we obtained SSR values of 48.0 and 19.9 for the INS and the ONS conditions, respectively. Both values pass the statistical test of goodness of fit (Additional File 1: Table S8). The final model configuration is shown in Additional File 2: Fig. S2. The best fit flux results for both metabolic growth conditions are listed in detail in Additional File 1: Table S9.

When net and exchange fluxes around the CO2_RBC pool were expressed based on the forward/backward formulation (Additional File 2: Fig. S1D), it was found that under the INS condition 58 ± 7% of CO2_RBC derives from atmospheric unlabeled CO_2_ and 42 ± 7% is produced by decarboxylation activities in metabolism that produce ^13^C-labeled CO_2_. Under the ONS condition 60 ± 7% of CO2_RBC derives from atmospheric CO_2_ (Additional File 2: Fig. S1D). The best fit flux solutions therefore are consistent with the idea of fast access of atmospheric CO_2_ to the RuBisCO sites.

### Statistical evaluation of flux results

The statistical uncertainty of fluxes that are derived from iterative fitting of the isotopomer model to ^13^C-labeling data was derived by a Monte Carlo approach using the 13CFLUX2 software tool [24]. For each condition, the original labeling data and uptake flux values were corrupted 20 times by adding pseudo-random noise to the ^13^C-labeling data according to the standard deviations [69]. Each time the best fit flux solution was selected from repeated optimization starting from 50 random start points for the free fluxes. The resulting 20 sets of best fit fluxes were used to derive the statistical uncertainty in the flux values (standard deviation). For the INS condition, the covariance matrix of net fluxes showed that the highest statistical uncertainty is between reactions of the pentose phosphate pathway and glycolysis, which occur in parallel in the cytosol and the plastid. It appears that some aspects of subcellular distribution of flux are not well resolved given the labeling data that are available. Therefore, to avoid over-interpretation of the results, the flux results were aggregated into a mostly non-compartmentalized view of the metabolic network. In essence, flux values for reactions between pairs of metabolites that are present in different subcellular compartments are added to obtain the rate of the merged reaction. At the same time, transport reactions between metabolic pools that were merged disappear. The same numeric operations are applied to both the best fit flux vectors and to the Monte Carlo sampled flux vectors. This way the statistical uncertainty of the combined reactions can be determined. Similar numerical operations were also applied to derive flux ratios or other linear combinations between fluxes of interest along with their accompanied statistical uncertainties.

### Analysis of chloroplasts distribution using Confocal Laser Scanning Microscopy (CLSM)

Living plants of *L. gibba* were analyzed non-invasively using the computer-assisted Zeiss LSM 780 CLSM (Carl Zeiss, Jena, Germany) and the commercial software package ZEN [101]. The helium–neon laser line 633 nm was applied for excitation. Auto-fluorescence of chlorophyll was detected with a 650 nm long-pass filter. The 3D in-depth imaging and 3D reconstruction of the chloroplast distribution in living cells was based on the optical serial sections, which were generated in Z-direction starting from the surface of the intact frond. In total, 133 sections (slice thickness 0, 36 µm) were integrated for the animated presentation.

### Statistical analysis

Comparisons between two metabolic growth conditions were performed using one-way ANOVA with 3 or 4 biological replicates per condition (as indicated in the Fig. or table legends). Unless stated otherwise, mean values were deemed to be statistically significantly different for *p* < 0.05.

### Supplementary Information

**Additional file 1: Table S1.** Increase in fronds area (cm^2^) during culture time (hours). Means and standard deviations represented three independent replicates. **Table S2.** Metabolite analysis by Liquid Chromatography / Mass Spectrometry. See also Table S13. **Table S3.** Biomass composition data (weight % in dry weight).  Means and standard deviations represented three independent replicates. **Table S4.** Measurements of ^13^C-enrichment in metabolites by GC/MS. **Table S5.** Measurement of ^13^C-enrichment in total frond biomass by Isotope-Ratio Mass Spectrometry. *L. gibba* fronds were grown with medium glucose consisting of 40% U^13^C_6_ glucose (99% ^13^C) and 60% unlabeled glucose (1.1% natural ^13^C abundance). **Table S6.** Definition of carbon flux model with indication of change in gene expression and change in flux. **Table S7.** Metabolite names used in the flux model. **Table S8.** Assessment of step wise model improvement. The four model configurations (A, B, C) correspond panels A, B and C in Fig. S1, respectively. **Table S9.** Best fit flux results for inorganic nitrogen condition (INS) and organic nitrogen condition (ONS). **Table S10.** Flux values with statistical confidence measures. **Table S11.** Differential expression analysis data (Outputs of DESeq package, A= INS condition, B= ONS condition). **Table S12.** List of Uniprot accession numbers associated with *Lemna gibba* protein IDs (information retrieved on April 4, 2018, http://www.uniprot.org). **Table S13.** GO term enrichment analysis for significantly differently expressed genes of *Lemna gibba*. **Table S14.** Mass spectrometer parameter settings for analysis of medium substrate levels. **Table S15.** Mass spectrometer parameter settings for metabolites measurements. Liquid chromatography coupled to mass spectrometer. **Table S16.** Primer sequences for qRT-PCR.

**Additional file 2: Fig. S1.** Schematics for different model configurations for which the best fit between ^13^C-MFA simulation and experimental data was determined. **Fig. S2.** Central metabolism network of growing Lemna gibba fronds. **Fig. S3.** Central metabolism Flux map of growing *Lemna gibba* fronds. **Fig. S4.** Statistical uncertainty in the flux values for the oxidative steps of the Oxidative Pentose Phosphate Pathway (OPPP). **Fig. S5.** Comparison the relative gene expression level by DEseq and quantitative real-time reverse transcription PCR (qRT-PCR).

**Additional file 3. **

**Additional file 4. **

## Data Availability

RNA sequencing data can be found in the Gene Expression Omnibus (GEO) under the accession GSE153602. All data in this study are available from the corresponding author upon reasonable request.

## Abbreviations

AcCoA: Acetyl-Coenzyme A
^13^C-MFA: ^13^C-Metabolic flux analysis
CBB: Calvin-Benson-Bassham
Cit: Citrate
CLSM: Confocal Laser Scanning Microscopy
DAVID: Database for Annotation: Visualization and Integrated Discovery
DESeq: Differential gene expression analysis of RNA-seq data
E2P2: Ensemble Enzyme Prediction Pipeline
EP: Erythrose 4-phosphate
FAMEs: Fatty acid methyl esters
GC/MS: Gas chromatography/mass spectrometry
GO: Gene ontology
GOGAT: Glutamine Oxoglutarate Aminotransferase
HP: Hexose phosphate
Icit: Isocitrate
INS: Inorganic nitrogen source
KG: α-Ketoglutarate
MAAs: Methyl ester alditol acetates
Mal: Malate
MFA: Metabolic flux analysis
MSTFA: N-Methyl-N-(trimethylsilyl) trifluoroacetamide
OAA: Oxaloacetate
ONS: Organic nitrogen source
OPPP: Oxidative pentose-phosphate pathway
PEP: Phosphoenol pyruvate
PGA: 3-Phosphoglycerate
Pyr: Pyruvate
RP: Ribose 5-phosphate
qPCR: Quantitative polymerase chain reaction
qRT-PCR: Quantitative real-time polymerase chain reaction
RNA-seq: RNA sequencing
RuBisCO: Ribulose-1:5-bisphosphate carboxylase/oxygenase
RuP: Ribulose 5-phosphate
SH medium: Schenk and Hildebrandt Basal Salt Mixture medium
SIM: Select ion monitor
SP: Sedoheptulose 7-phosphate
Succ: Succinate
TCA: Tricarboxylic acid
TFAA: Trifluoroacetic acid anhydride
TP: Triose phosphate (dihydroxyacetone phosphate, glyceraldehyde 3-phosphate)
UCD SIF: University of California Davis Stable Isotope Facility
XP: Xylulose 5-phosphate

## Footnotes

1 Biosynthetic products are expected to be labeled close to the average ^13^C-enrichment in medium glucose (12.3%). However, due to the unequal distribution of ^13^C in the carbon chain of [1-^13^C]-glucose, this value changes to the extent that glucose carbons do not contribute equally to a biosynthetic product.
