# Supplementary material for: Mechanisms of metabolic adaptation in the duckweed Lemna gibba: an integrated metabolic, transcriptomic and flux analysis
Source: BMC Plant Biol. 2023 Oct 3;23:458. doi: 10.1186/s12870-023-04480-9 (PMC10546790; doi:10.1186/s12870-023-04480-9)
Supplement: Supplementary file 2 — Additional file 2: Fig. S1. Schematics for different model configurations for which the best fit between 13C-MFA simulation and experimental data was determined. Fig. S2. Central metabolism network of growing Lemna gibba fronds. Fig. S3. Central metabolism Flux map of growing Lemna gibba fronds. Fig. S4. Statistical uncertainty in the flux values for the oxidative steps of the Oxidative Pentose Phosphate Pathway (OPPP). Fig. S5. Comparison the relative gene expression level by DEseq and quantitative real-time reverse transcription PCR (qRT-PCR). [file 12870_2023_4480_MOESM2_ESM.pdf]

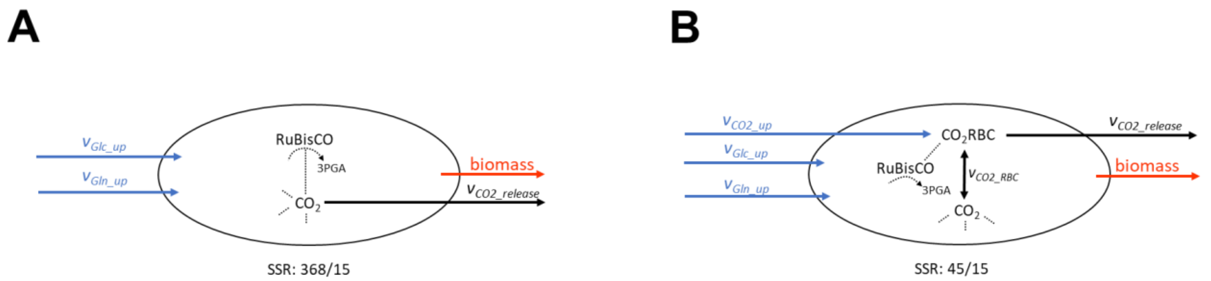

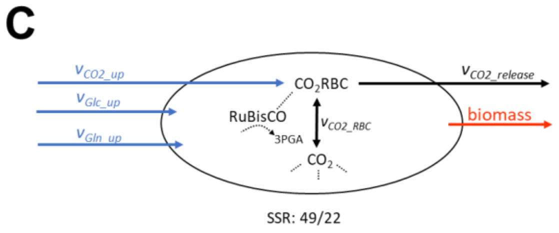

Flux name in model	description	INS	ONS
vGlc_up	Glucose uptake	102.27 ± 4.36	80.32 ± 2.48
vGln_up	Gln uptake	0	79.58 ± 2.09
vCO2_up	CO_2 uptake	589.7 ± 79.3	521.8 ± 56.4

(μmol·g-dw^{-1}·h^{-1})

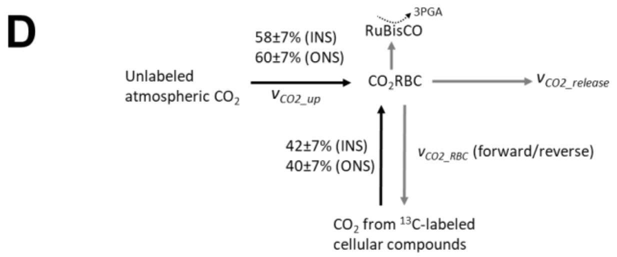

Additional File 2, Fig. S1: Schematics for different model configurations for which the best fit between ^{13}C-MFA simulation and experimental data was determined. Red arrows represent fixed rates. Blue arrows denote flux defined as free adjustable in the flux model. **A**, no CO_2 uptake possible. **B**, a CO_2 uptake was added together with a distinct CO_2 pool that is only accessible by RuBisCO. Reaction vCO2_RBC has a freely adjustable reversibility component. Panel **C** shows a model configuration together with flux values added as measurements into the model (Table 2, main text). **D**, For the flux solution of panel C, fluxes around CO2_RBC are shown in the forward/reverse formulation. The relative influx into the CO2_RBC pool are given. SSR: Sum of Squares of weighted Residuals. To pass the test for goodness of fit, the SSR needs to be below about 60 (Slightly differs with model configurations, see Additional File 1: Table S8). Abbreviations: 3PGA, 3-phosphoglycerate.

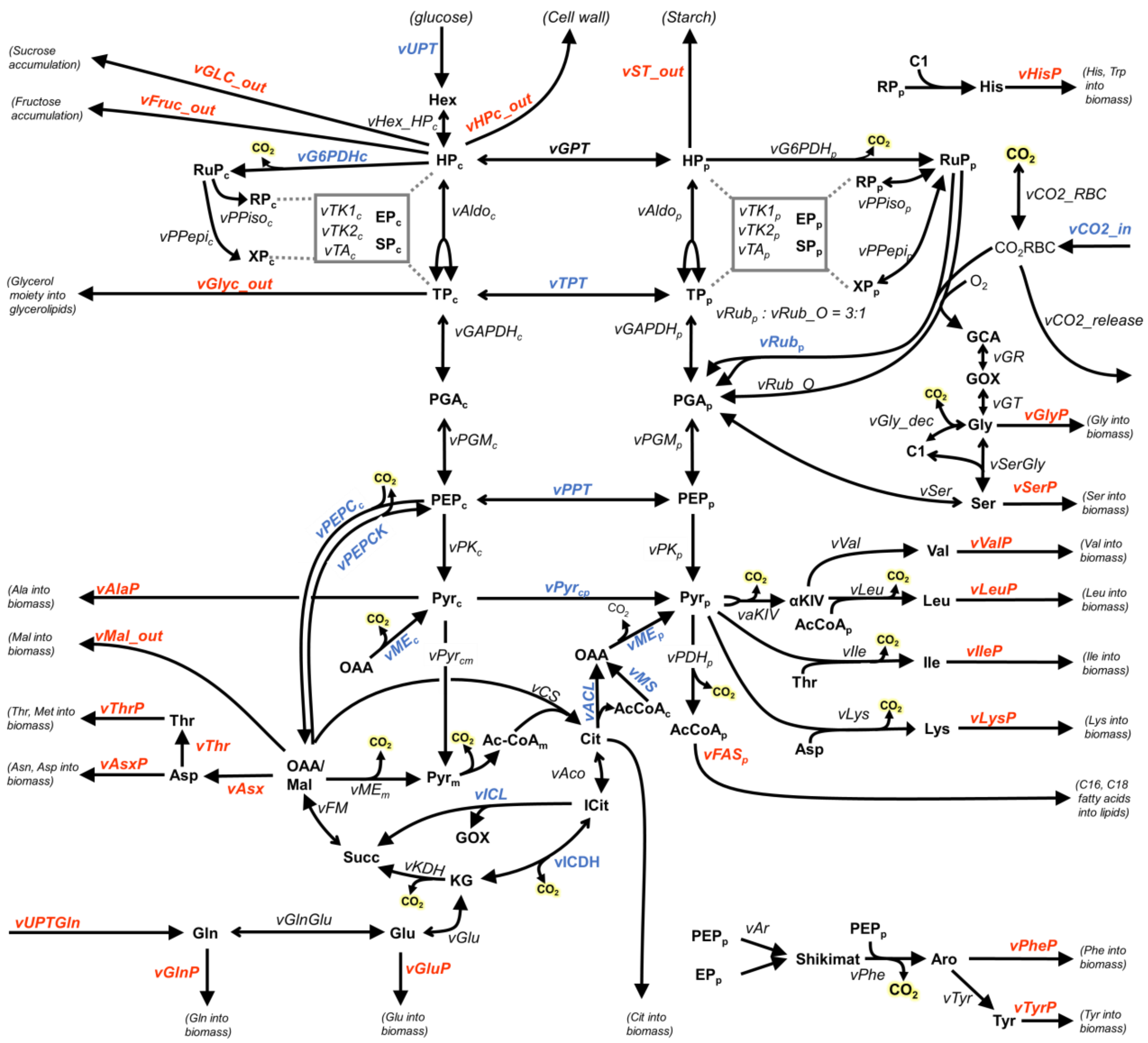

Additional File 2, Fig. S2: Central metabolism network of growing *Lemna gibba* fronds. Scheme of the general network structure. Blue font, free net fluxes; red font, flux values directly derived (fixed) by measurements of growth rate and biomass composition. For reactions can proceed in both directions there are two arrowheads and the direction of positive flux values is represented by the larger arrowhead. Subscripts c, p, m denote cytosol, plastid, mitochondrial compartment. See Table S6 for abbreviations and more details on the reactions.

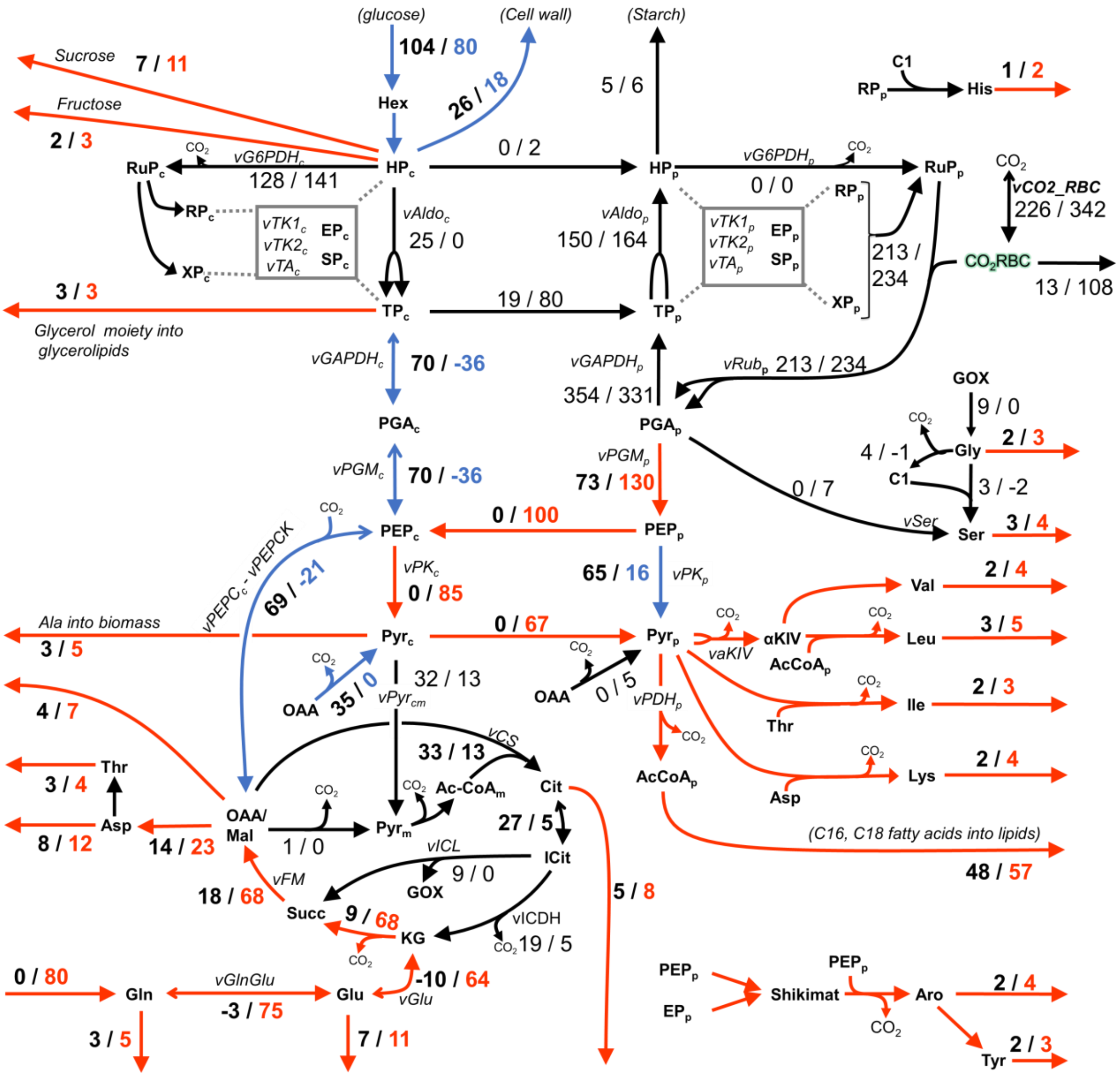

Additional File 2, Fig. S3: Central metabolism Flux map of growing *Lemna gibba* fronds. Scheme of the general network structure (absolute flux values, µmol·g-DW^{-1}·hr^{-1}). Flux values for the two nitrogen conditions are shown together (INS flux / ONS flux). Red (blue) arrows indicate that absolute flux was significantly higher under the INS or ONS condition, respectively. For reactions that proceed in different directions the direction of the INS flux is represented by the larger arrowhead. All flux values with statistical confidence measures are listed in Table S10.

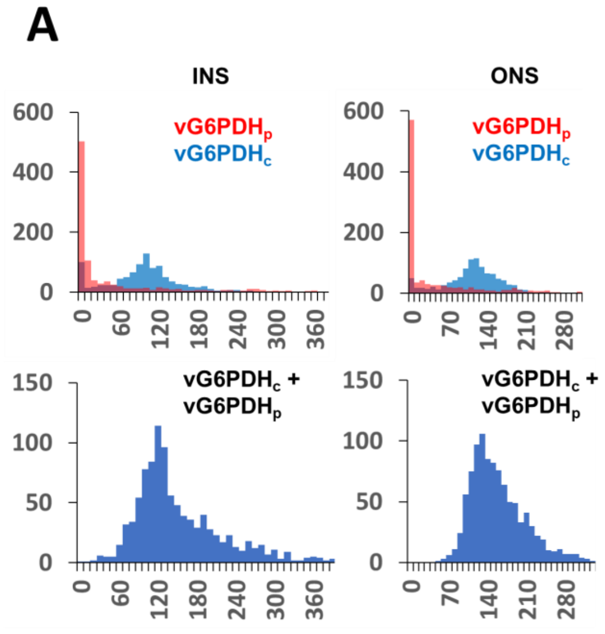

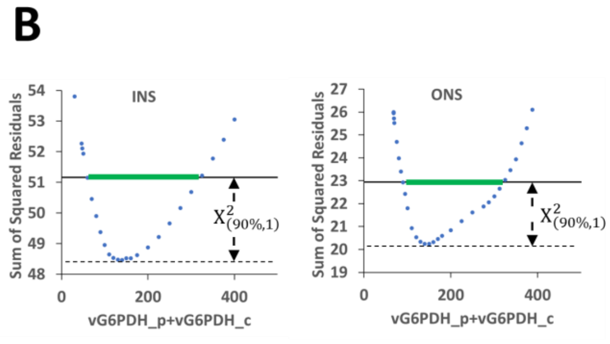

C

condition	Flux	90% confidence interval
INS	vG6PDH_c	[0.00, 203.39]
	vG6PDH_p	[0.00, 288.49]
	vG6PDH_c + vG6PDH_p^S	[9.04, 399.79]
	vG6PDH_c + vG6PDH_p	[74.71, 309.35]
	vG6PDH_c + vG6PDH_p (p.c.)	[60.24, 322.14]
ONS	vG6PDH_c	[10.57, 195.60]
	vG6PDH_p	[0.00, 225.87]
	vG6PDH_c + vG6PDH_p^S	[28.40, 353.21]
	vG6PDH_c + vG6PDH_p	[102.01, 271.95]
	vG6PDH_c + vG6PDH_p (p.c.)	[89.71, 322.32]

S: the order of one vector was scrambled before adding the values.

Additional File 2, Fig. S4: Statistical uncertainty in the flux values for the oxidative steps of the Oxidative Pentose Phosphate Pathway (OPPP). Statistics for OPPP flux through cytosol (c) and plastid (p), are assessed in separate and when summed, which constitutes the combined OPPP flux. **A**, Monte Carlo approach. The model was re-fitted 1000 times, each time after perturbing the measurement data by adding random noise. Shown is the distribution of OPPP flux values. **B**, 90 % confidence intervals for the combined cytosolic and plastidic OPPP fluxes were also determined by repeated re-optimization of the model when the sum of the fluxes vG6PDHc and vG6PDHp was constrained to a fixed value each time (parameter continuation). Determination of the 90% confidence intervals according to Antoniewicz et al., Metab. Eng. 2006, 8: 324-337. **C**, 90% confidence intervals based on Monte Carlo statistics and the parameter continuation strategy (green). For both methods, the resulting 90 % confidence intervals for the combined OPPP fluxes are very similar. The combined OPPP fluxes can therefore be assumed to be above 60 and 90 for the INS and ONS condition, respectively. As a result of joint statistical distribution, the sum of fluxes (vG6PDH_c + vG6PDH_p) is less dispersed than vG6PDH_c and vG6PDH_p. This effect is reduced when the addition of flux samples is repeated after randomly changing the order of one of the value sets ("S").

For the Monte-Carlo simulations shown in this figure the modelling tool **influx_s** (Sokol S, Millard P, Portais J-C, Bioinformatics 2011, 28(5):687-693.), version 6.1., was used with the parameters "--ln --noscale --irand --sens=mc=1000".

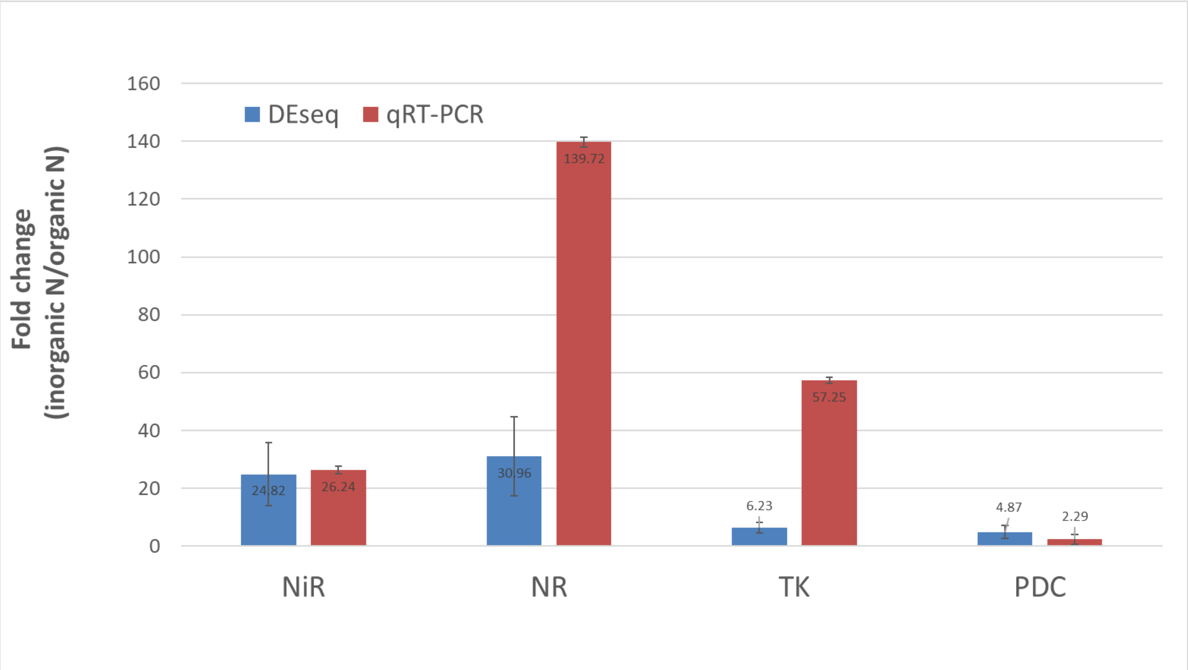

Additional File 2, Fig. S5: Comparison the relative gene expression level by DEseq and quantitative real-time reverse transcription PCR (qRT-PCR). Data are means ± SD (n=3). Primer sequences see Table S16. Abbreviations: NiR, Ferredoxin-nitrite reductase, chloroplastic (EC 1.7.7.1), LEMGIv51_A5_006998_1; NR, Nitrate reductase [NADH] (NR) (EC 1.7.1.1), LEMGIv51_A5_009812_1; TK, Transketolase, chloroplastic (TK) (EC 2.2.1.1), LEMGIv51_A5_017459_1; PDC, Pyruvate decarboxylase (EC 4.1.1.1), LEMGIv51_A5_020915_1.
